# Prognostication of patients with clear cell renal cell carcinomas based on quantification of DNA methylation levels of CpG island methylator phenotype marker genes

**DOI:** 10.1186/1471-2407-14-772

**Published:** 2014-10-20

**Authors:** Ying Tian, Eri Arai, Masahiro Gotoh, Motokiyo Komiyama, Hiroyuki Fujimoto, Yae Kanai

**Affiliations:** Division of Molecular Pathology, National Cancer Center Research Institute, 5-1-1 Tsukiji, Chuo-ku, Tokyo, 104-0045 Japan; Department of Urology, National Cancer Center Hospital, Tokyo, 104-0045 Japan

**Keywords:** DNA methylation, CpG island methylator phenotype (CIMP), Prognostication, MassARRAY system, Clear cell renal cell carcinoma (ccRCC)

## Abstract

**Background:**

The CpG island methylator phenotype (CIMP) of clear cell renal cell carcinomas (ccRCCs) is characterized by accumulation of DNA methylation at CpG islands and poorer patient outcome. The aim of this study was to establish criteria for prognostication of patients with ccRCCs using the ccRCC-specific CIMP marker genes.

**Methods:**

DNA methylation levels at 299 CpG sites in the 14 CIMP marker genes were evaluated quantitatively in tissue specimens of 88 CIMP-negative and 14 CIMP-positive ccRCCs in a learning cohort using the MassARRAY system. An additional 100 ccRCCs were also analyzed as a validation cohort.

**Results:**

Receiver operating characteristic curve analysis showed that area under the curve values for the 23 CpG units including the 32 CpG sites in the 7 CIMP-marker genes, i.e. *FAM150A, ZNF540, ZNF671, ZNF154, PRAC, TRH and SLC13A5,* for discrimination of CIMP-positive from CIMP-negative ccRCCs were larger than 0.95. Criteria combining the 23 CpG units discriminated CIMP-positive from CIMP-negative ccRCCs with 100% sensitivity and specificity in the learning cohort. Cancer-free and overall survival rates of patients with CIMP-positive ccRCCs diagnosed using the criteria combining the 23 CpG units in a validation cohort were significantly lower than those of patients with CIMP-negative ccRCCs (*P* = 1.41 × 10^−5^ and 2.43 × 10^−13^, respectively). Patients with CIMP-positive ccRCCs in the validation cohort had a higher likelihood of disease-related death (hazard ratio, 75.8; 95% confidence interval, 7.81 to 735; *P* = 1.89 × 10^−4^) than those with CIMP-negative ccRCCs.

**Conclusions:**

The established criteria are able to reproducibly diagnose CIMP-positive ccRCCs and may be useful for personalized medicine for patients with ccRCCs.

**Electronic supplementary material:**

The online version of this article (doi:10.1186/1471-2407-14-772) contains supplementary material, which is available to authorized users.

## Background

Clear cell renal cell carcinoma (ccRCC) is the most common histological subtype of adult kidney cancer [1]. In general, ccRCCs at an early stage are curable by nephrectomy. However, some ccRCCs relapse and metastasize to distant organs, even if the resection has been considered complete [2]. Even though novel targeting agents have been developed for treatment of ccRCC, unless relapsed or metastasized tumors are diagnosed early by close follow-up, the effectiveness of any therapy is restricted [3]. Therefore, reliable prognostic criteria need to be established.

Not only genetic, but also epigenetic events appear to accumulate during carcinogenesis, and DNA methylation alterations are one of the most consistent epigenetic changes in human cancers [4–6]. We and other groups have revealed that DNA methylation alterations participate in renal carcinogenesis and are significantly correlated with the clinicopathological diversity of ccRCCs [7–11]. In addition, a distinct cancer phenotype known as the CpG island methylator phenotype (CIMP), characterized by accumulation of DNA methylation at CpG islands, has been defined in well-studied cancers [12, 13] such as those of the colorectum [14] and stomach [15], and shown to be significantly correlated with clinicopathological parameters. Although the relevance of the CIMP-positive phenotype in the context of ccRCCs has not yet been clearly defined [16], our group very recently identified CIMP-positive ccRCCs based on genome-wide DNA methylation analysis [7]. We also identified 17 genes, i.e. *FAM150A, GRM6, ZNF540, ZFP42, ZNF154, RIMS4, PCDHAC1, KHDRBS2, ASCL2, KCNQ1, PRAC, WNT3A, TRH, FAM78A, ZNF671, SLC13A5* and *NKX6-2*, which are hallmarks of CIMP in ccRCCs [7], using single CpG-resolution Infinium assay [17]. The CIMP-positive ccRCCs in our cohort were clinicopathologically aggressive and associated with poorer patient outcome [7], indicating that CIMP in ccRCCs might be applicable as a prognostic indicator.

However, in our previous study, CIMP-positive ccRCCs were identified using hierarchical clustering analysis based on DNA methylation profiles in the examined cohort [7]. The DNA methylation status of entire promoter CpG islands, other than Infinium probe sites, in the CIMP marker genes has not been evaluated quantitatively. Therefore, to establish criteria for CIMP diagnosis that would be applicable to individual patients, CpG sites having the largest diagnostic impact should be identified in the entire promoter CpG islands of the CIMP marker genes based on quantification of DNA methylation levels. Moreover, appropriate cutoff values of DNA methylation levels need to be established for the identified CpG sites in order to discriminate CIMP-positive from CIMP-negative ccRCCs.

In the present study, we quantitatively evaluated DNA methylation levels at 299 CpG sites throughout the promoter CpG islands of the ccRCC-specific CIMP marker genes in 88 CIMP-negative ccRCCs and 14 CIMP-positive ccRCCs using the MassARRAY system. We then validated the prognostic impact of the established criteria for CIMP diagnosis in a validation cohort of 100 additional ccRCCs.

## Methods

### Patients and tissue samples

As a learning cohort, 102 samples of cancerous tissue obtained from specimens surgically resected from 102 patients with primary ccRCCs were subjected to the present analysis. These patients did not receive preoperative treatment and underwent nephrectomy at the National Cancer Center Hospital, Tokyo, Japan. There were 71 men and 31 women with a mean (± standard deviation) age of 62.9 ± 10.4 years (range, 36 to 85 years). Histological diagnosis was made in accordance with the World Health Organization classification [18].

In our previous study, unsupervised hierarchical clustering based on genome-wide DNA methylation analysis using single CpG-resolution Infinium assay divided the 102 ccRCCs in the learning cohort into 88 CIMP-negative ccRCCs and 14 CIMP-positive ccRCCs [7]. In the same study, we showed that the CIMP-positive ccRCCs were clinicopathologically more aggressive and associated with a poorer patient outcome than CIMP-negative ccRCCs [7]: the clinicopathological characteristics [19, 20] of CIMP-negative and CIMP-positive ccRCCs in the learning cohort are summarized in Additional file 1: Table S1.

As a validation cohort, 100 samples of cancerous tissue were obtained from specimens surgically resected from 100 patients with primary ccRCCs. These patients also did not receive preoperative treatment and underwent nephrectomy at the National Cancer Center Hospital, Tokyo, Japan. The patients comprised 68 men and 32 women with a mean (± standard deviation) age of 62.5 ± 11.4 years (range, 33 to 87 years). The clinicopathological characteristics [19, 20] of ccRCCs in the validation cohort are summarized in Additional file 2: Table S2.

Tissue specimens were taken and frozen immediately after surgical removal and have been stored in liquid nitrogen until DNA extraction. ccRCCs are hypervascular tumors with an increased opportunity for infiltration of non-cancerous cells such as lymphocytes [21]: the microscopically examined tumor cell contents (%) of all ccRCC tissue specimens in the learning and validation cohorts are shown in Additional file 3: Table S3. Tissue specimens were provided by the National Cancer Center Biobank, Tokyo, Japan. This study was approved by the Ethics Committee of the National Cancer Center, Tokyo, Japan, and was performed in accordance with the Declaration of Helsinki. All the patients provided written informed consent prior to inclusion in the study.

### DNA extraction and bisulfite modification

High-molecular-weight DNA was extracted from fresh-frozen tissue samples using phenol-chloroform followed by dialysis [22]. One microgram of genomic DNA was subjected to bisulfite treatment using an EpiTect Bisulfite Kit (QIAGEN GmbH, Hilden, Germany), in accordance with the manufacturer’s protocol. This process converts non-methylated cytosine to uracil, while methylated cytosine remains unchanged [23].

### Quantitative DNA methylation analysis with the MassARRAY system

DNA methylation levels at individual CpG sites were evaluated quantitatively using the MassARRAY platform (Sequenom, San Diego, CA). This method utilizes base-specific cleavage and matrix-assisted laser desorption/ionization time-of-flight mass spectrometry (MALDI-TOF MS) [24]. Specific PCR primers for bisulfite-converted DNA were designed using the EpiDesigner software package (http://www.epidesigner.com, Sequenom), encompassing all promoter CpG islands of the previously identified ccRCC-specific CIMP marker genes [7]. The sequences of the 16 primer sets are given in Additional file 4: Table S4. A T7-promoter tag (5′-CAGTAATACGACTCACTATAGGGAGAAGGCT-3′) was added to each reverse primer for in vitro transcription, and a 10-mer tag (5′-AGGAAGAGAG-3′) was added to each forward primer to balance the PCR.

To overcome PCR bias in DNA methylation analysis, we optimized the annealing temperature and type of DNA polymerase: 0%, 50% and 100% methylated control DNA (Epitect methylated human control DNA; QIAGEN) was used as template to test the linearity of the protocol. Using HotStar Taq DNA polymerase (QIAGEN) or TaKaRa Taq HS DNA polymerase (Takara Bio, Shiga, Japan), the annealing temperature for each of the 16 primer sets was set to give a correlation coefficient (R^2^) of more than 0.9 and to make the slope of the standard curve close to 1 (Additional file 5: Figure S1 and Additional file 4: Table S4). The PCR products were separated electrophoretically on 2% agarose gel and stained with ethidium bromide to confirm that specific products of the appropriate size and no non-specific products were obtained upon amplification.

Then, the PCR products were used as a template for in vitro transcription and the RNase A-mediated cleavage reaction using an EpiTYPER Reagent Kit (Sequenom). The fragmented samples were dispensed onto a SpectroCHIP array, and then detected on a MassARRAY analyzer compact MALDI-TOF MS instrument. The data were visualized using EpiTYPER Analyzer software v1.0 (Sequenom). The DNA methylation level (%) at each CpG site was determined by comparing the signal intensities of methylated and non-methylated templates. A cluster of consecutive CpG sites, each giving one measured value by the MassARRAY system, is defined as a “CpG unit” in the manufacturer’s protocol. The DNA methylation levels at the 299 examined CpG sites in the CIMP marker genes were then expressed as data for the 193 CpG units. Experiments were performed in triplicate for each sample-CpG unit, and the mean value for the three experiments was used as the DNA methylation level.

### Statistics

Differences in DNA methylation levels at individual CpG units between CIMP-positive ccRCCs and CIMP-negative ccRCCs were analyzed using Mann–Whitney *U* test. The CpG units having the largest diagnostic impact were identified by receiver operating characteristic (ROC) curve analysis [25]: For 23 CpG units showing area under the curve (AUC) values larger than 0.95, appropriate cutoff values were determined in order to discriminate CIMP-positive from CIMP-negative ccRCCs [26]. For discriminating CIMP-positive from CIMP-negative ccRCCs, the Youden index [26] was used as a cutoff value for each CpG unit. Survival curves for patients with ccRCCs were analyzed by the Kaplan-Meier method and the log-rank test. Correlations between DNA methylation levels and recurrence and disease-related death were analyzed using the Cox proportional hazards model. All statistical analyses were performed using SPSS statistics version 20 (IBM Corp., Armonk, NY). Differences at *P* values of less than 0.05 were considered statistically significant.

## Results

### DNA methylation status of CIMP marker genes in CIMP-negative and CIMP-positive ccRCCs

Previously, we had identified 17 ccRCC-specific CIMP marker genes based on genome-wide DNA methylation analysis using the Infinium HumanMethylation27K BeadChip [7]. Six exact Infinium probe CpG sites in ccRCC-specific CIMP marker genes (Probe ID: cg06274159 for the *ZFP42* gene, cg03975694 for the *ZNF540* gene, cg08668790 for the *ZNF154* gene, cg01009664 for the *TRH* gene, cg22040627 for the *SLC13A5* gene, and cg19246110 for the *ZNF671* gene) were examined using the MassArray system in the learning cohort (Additional file 6; Figure S2). Significant correlations between DNA methylation levels determined by our previous Infinium assay [7] and those determined by the present MassArray analysis were statistically confirmed (*P* = 1.25 × 10^−35^, *P* = 1.98 × 10^−32^, *P* = 1.31 × 10^−41^, *P* = 5.30 × 10^−34^, *P* = 7.91 × 10^−22^ and *P* = 7.61 × 10^−44^, respectively).

**Figure 1:**
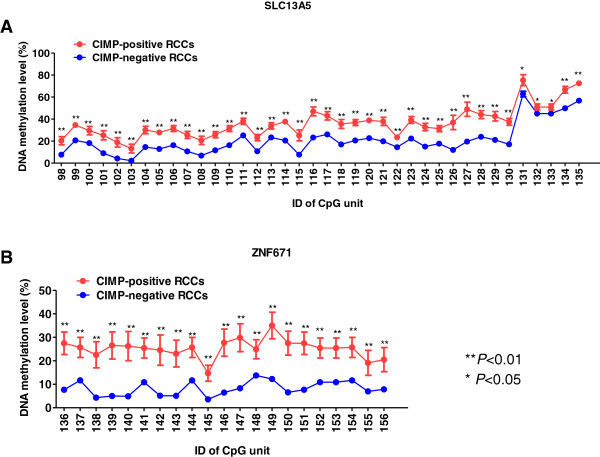
**Average DNA methylation levels at promoter CpG islands in the** ***SLC13A5*** **(A) and** ***ZNF671*** **(B) genes in CIMP-negative (n = 88) and CIMP-positive (n = 14) ccRCCs in the learning cohort.** DNA methylation levels of each CpG unit were evaluated quantitatively using the MassARRAY system. **A**. Average DNA methylation levels of all examined 38 CpG units including 68 CpG sites located within 1347 bp 5′ region of the *SLC13A5* gene in CIMP-positive ccRCCs (red line) were significantly higher than those in CIMP-negative ccRCCs (blue line). **B**. Average DNA methylation levels of all examined 21 CpG units including 29 CpG sites located within the 428 bp 5′ region of the *ZNF671* gene in CIMP-positive ccRCCs (red line) were significantly higher than those in CIMP-negative ccRCCs (blue line). *P < 0.05 and **P < 0.01. Exact *P* values for each CpG unit of the *SLC13A5* and *ZNF671* genes are summarized in Additional file 7: Table S5. Error bar: standard error.

In the present study, our primary intention was to evaluate quantitatively the DNA methylation status of not only the Infinium probe sites but also the entire promoter CpG islands in the ccRCC-specific CIMP marker genes using the MassARRAY system [24]. Since the promoter regions of the CIMP marker genes, *KCNQ1, FAM78A* and *NKX6-2*, have a very high GC content, for these three genes we were unable to set optimized PCR conditions. Then, the DNA methylation status of 193 CpG units including 299 CpG sites in the remaining 14 ccRCC-specific CIMP marker genes, i.e. *FAM150A, GRM6, ZNF540, ZFP42, ZNF154, RIMS4, PCDHAC1, KHDRBS2, ASCL2, PRAC, WNT3A, TRH, ZNF671* and *SLC13A5*, was evaluated quantitatively using the MassARRAY system. The average DNA methylation levels of 38 CpG units including 68 CpG sites located within the 1347 bp 5′-region of the representative CIMP marker gene, *SLC13A5,* in CIMP-negative (n = 88) and CIMP-positive (n = 14) ccRCCs in the learning cohort are shown in Figure 1A. Similarly, the average DNA methylation levels of 21 CpG units including 29 CpG sites located within the 428 bp 5′-region of another representative CIMP marker gene, *ZNF671*, in CIMP-negative and CIMP-positive ccRCCs in the learning cohort are shown in Figure 1B. The average DNA methylation levels of all the CpG units examined (59 in total) in the *SLC13A5* and *ZNF671* genes in the CIMP-positive ccRCCs were significantly higher than those in CIMP-negative ccRCCs (the *P* values for each CpG unit are shown in Additional file 7: Table S5). Similarly, the average DNA methylation levels of 130 CpG units including 195 CpG sites, out of the 134 CpG units examined including 202 CpG sites in the remaining 12 CIMP marker genes, in the CIMP-positive ccRCCs were significantly higher than those in CIMP-negative ccRCCs (Additional file 7: Table S5). These data indicated that almost the entire promoter CpG islands in all the CIMP marker genes examined were methylated in CIMP-positive ccRCCs.

### Establishment of criteria for discriminating CIMP-positive from CIMP-negative ccRCCs in the learning cohort

Since quantitative DNA methylation analysis using the MassARRAY system revealed that many CpG sites showed significant differences in DNA methylation levels between CIMP-negative and CIMP-positive ccRCCs among all the promoter CpG islands of CIMP marker genes (Figure 1 and Additional file 7: Table S5), we attempted to identify CpG sites having the largest diagnostic impact, and to establish criteria for discriminating CIMP-positive from CIMP-negative ccRCCs. ROC curves were constructed for all 193 CpG units examined, including 299 CpG sites in the 14 CIMP marker genes examined, and the corresponding AUC values [25] were calculated. Eighty-six CpG units, including 135 CpG sites, showed AUC values larger than 0.9 (Additional file 8: Table S6). Among these 86, the top 23 CpG units including 32 CpG sites showing AUC values larger than 0.95 were used to establish the criteria for discriminating CIMP-positive from CIMP-negative ccRCCs (Table 1). For discriminating CIMP-positive from CIMP-negative ccRCCs, the Youden index [26] was used as a cutoff value for each CpG unit (Table 1).

**Table 1:** **The 23 CpG units showing area under the curve (AUC) values larger than 0.95 in receiver operating characteristic curve analysis for discrimination of CpG island methylator phenotype (CIMP)-positive clear cell renal cell carcinomas (ccRCCs) from CIMP-negative ccRCCs in the learning cohort**

ID of CpG unit ^1^	Gene symbol	Chromo- some	Position of CpG site ^2^	AUC value	Cutoff value ^3^(%)	Sensitivity ^4^ (%)	Specificity ^4^ (%)
81	*TRH*	3	129693406, 129693412	0.973	30.8	100.0	88.6
85	*TRH*	3	129693518, 129693521, 129693528	0.950	18.2	100.0	78.4
89	*TRH*	3	129693586	0.952	11.0	92.3	92.0
94	*TRH*	3	129693635	0.967	6.6	100.0	87.5
8	*FAM150A*	8	53478477	0.968	27.2	83.3	94.1
11	*FAM150A*	8	53478511	0.968	27.2	83.3	94.1
78	*PRAC*	17	46799755	0.957	40.7	92.9	89.8
102	*SLC13A5*	17	6616733	0.983	7.5	92.9	96.6
105	*SLC13A5*	17	6616812	0.983	18.5	100.0	94.3
106	*SLC13A5*	17	6616826, 6616828	0.951	23.3	100.0	88.6
107	*SLC13A5*	17	6616851, 6616854, 6616857	0.954	14.8	100.0	87.5
110	*SLC13A5*	17	6616927, 6616929	0.951	23.3	100.0	88.6
30	*ZNF540*	19	38042496	0.983	41.0	100.0	98.3
32	*ZNF540*	19	38042518	0.960	35.7	100.0	93.1
33	*ZNF540*	19	38042530, 38042532	0.991	36.4	100.0	96.6
43	*ZNF154*	19	58220567	0.956	13.3	92.9	90.9
44	*ZNF154*	19	58220627	0.966	14.8	85.7	95.5
45	*ZNF154*	19	58220657, 58220662	0.959	22.2	92.9	95.5
149	*ZNF671*	19	58238780	0.954	15.2	85.7	89.7
158	*ZNF671*	19	58238928	0.965	10.5	100.0	88.5
160	*ZNF671*	19	58238954	0.954	15.2	85.7	89.7
161	*ZNF671*	19	58238987	0.954	15.2	85.7	89.7
163	*ZNF671*	19	58239012	0.951	10.5	85.7	92.0

^1^ID of CpG unit is defined in Additional file 4: Table S4.

^2^National Center for Biotechnology Information (NCBI) Database (Genome Build 37).

^3^The Youden index was used as a cutoff value for discriminating CIMP-positive ccRCCs in the learning cohort from CIMP-negative ccRCCs. When the cancerous tissue shows a DNA methylation level equal to or higher than the cutoff value, the ccRCC is considered to be CIMP-positive; when the cancerous tissue shows a DNA methylation level lower than the cutoff value, the ccRCC is considered to be CIMP-negative.

^4^Sensitivity and specificity for discrimination of CIMP-positive ccRCCs in the learning cohort from CIMP-negative ccRCCs using individual CpG units.

**Figure 2:**
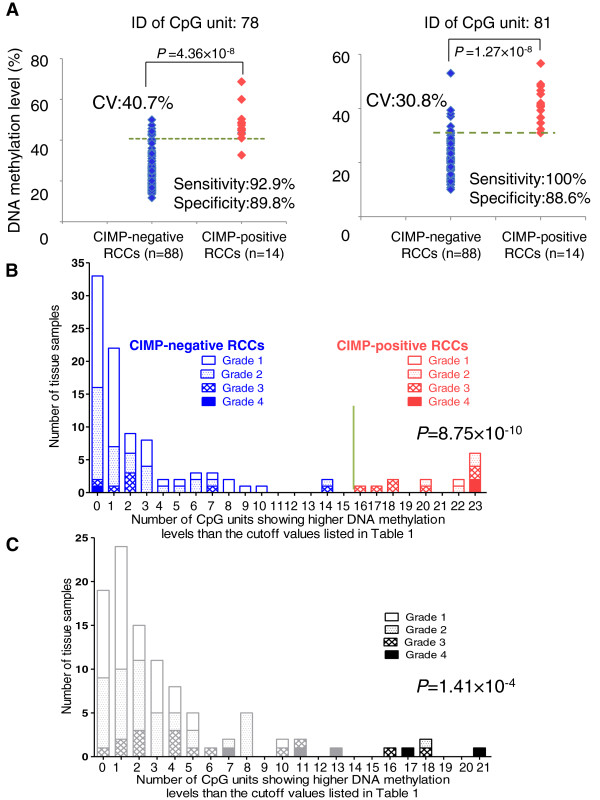
**The criteria for CIMP diagnosis discriminating CIMP-positive from CIMP-negative ccRCCs based on the MassARRAY system. A**. Scattergrams of DNA methylation levels of representative CpG units in the learning cohort. Using each CpG unit and its cutoff value (CV) described in Table 1, CIMP-positive ccRCCs were discriminated from CIMP-negative ccRCCs with sufficient sensitivity and specificity. **B**. Histogram showing the number of CpG units with DNA methylation levels higher than the cutoff values listed in Table 1 in the learning cohort. All 14 ccRCCs (red columns) showing DNA methylation levels higher than the cutoff values at 16 or more CpG units were CIMP-positive ccRCCs, and all 88 ccRCCs (blue columns) showing DNA methylation levels higher than the cutoff values at less than 16 CpG units were CIMP-negative ccRCCs. On the basis of this histogram, we established the following criteria: When the cancerous tissue showed DNA methylation levels higher than the cutoff values at 16 (green bar) or more CpG units, it was judged to be CIMP-positive. The number of CpG units showing higher DNA methylation levels than the cutoff values in CIMP-positive ccRCCs (20.79 ± 0.69) was higher than that of CIMP-negative ccRCCs (2.09 ± 0.32, *P* = 8.75 × 10^−10^). **C**. Histogram showing the number of CpG units with DNA methylation levels higher than the cutoff values listed in Table 1 in the additional 100 ccRCCs comprising the validation cohort. Using the criteria established on the basis of panel **B**, 5 ccRCCs (black bars) were diagnosed as CIMP-positive ccRCCs, whereas 95 ccRCCs (gray bars) were diagnosed as CIMP-negative ccRCCs. The number of CpG units showing higher DNA methylation levels than the cutoff values in ccRCCs diagnosed as CIMP-positive (18.00 ± 0.84) was higher than that of ccRCCs diagnosed as CIMP-negative (2.73 ± 0.30, *P* = 1.41 × 10^−4^).

Figure 2A shows scattergrams of the DNA methylation levels of representative CpG units in CIMP-negative and CIMP-positive ccRCCs in the learning cohort along with cutoff values listed in Table 1. The sensitivity and specificity of such discrimination using the cutoff values derived for each CpG unit are shown in Figure 2A and Table 1. A histogram showing the number of CpG units showing DNA methylation levels higher than the cutoff values listed in Table 1 in the learning cohort is shown in Figure 2B. All 14 ccRCCs showing DNA methylation levels higher than the cutoff values listed in Table 1 at 16 or more CpG units based on the present MassARRAY analysis (red bars in Figure 2B) were CIMP-positive ccRCCs identified by our previous hierarchical clustering based on the Infinium assay. All 88 ccRCCs showing DNA methylation levels higher than the cutoff values listed in Table 1 at less than 16 CpG units based on the present MassARRAY analysis (blue bars in Figure 2B) were CIMP-negative ccRCCs identified by our previous hierarchical clustering based on the Infinium assay.

Based on Figure 2B, we established the following criteria: when ccRCC tissue shows DNA methylation levels higher than the cutoff values listed in Table 1 at 16 or more CpG units (green line in Figure 2B), it is judged to be CIMP-positive. When ccRCC tissue shows DNA methylation levels higher than the cutoff values listed in Table 1 at less than 16 CpG units, it is judged to be CIMP-negative. Using these criteria, CIMP-positive ccRCCs in the learning cohort were discriminated from CIMP-negative ccRCCs with 100% sensitivity and specificity.

### Prognostic impact of CIMP diagnosis in the validation cohort

It has previously been revealed that patients with CIMP-positive ccRCCs show a poorer outcome [7]. Therefore, we attempted to validate the prognostic impact of CIMP diagnosis using criteria based on the cutoff values listed in Table 1. Using the additional 100 ccRCCs in the validation cohort, DNA methylation levels at the 23 CpG units including the 32 CpG sites in Table 1 were evaluated quantitatively using the MassARRAY system. The DNA methylation statuses of the 100 ccRCCs in the validation cohort were used to construct a histogram showing the number of CpG units with DNA methylation levels higher than the cutoff values listed in Table 1 (Figure 2C). The distribution of DNA methylation status at the 23 CpG units of the ccRCCs in the validation cohort (Figure 2C) was similar to that in the learning cohort (Figure 2B). Based on the criteria for CIMP diagnosis established using the learning cohort, 5 ccRCCs showing DNA methylation levels higher than the cutoff values listed in Table 1 at 16 or more CpG units were diagnosed as CIMP-positive, whereas 95 ccRCCs showing such higher DNA methylation levels at less than 16 CpG units were diagnosed as CIMP-negative.

**Figure 3:**
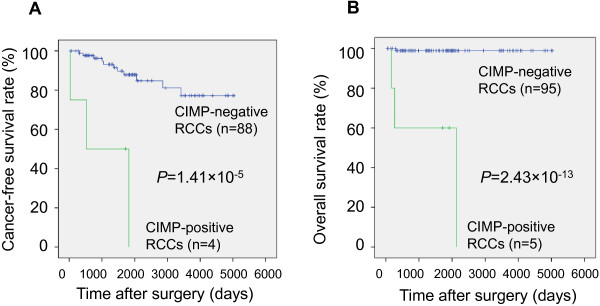
**Kaplan–Meier survival curves of patients with CIMP-positive and negative ccRCCs in the validation cohort.** Cancer-free (Panel **A**, *P* = 1.41 × 10^−5^) and overall (Panel **B**, *P* = 2.43 × 10^−13^) survival rates of patients with ccRCCs showing DNA methylation levels higher than the cutoff values listed in Table 1 at 16 or more CpG units (diagnosed as CIMP-positive ccRCCs) were significantly lower than those of patients with ccRCCs showing DNA methylation levels higher than the cutoff values listed in Table 1 at less than 16 CpG units (diagnosed as CIMP-negative ccRCCs). Patients who underwent curative resection were included in panel **A**. The prognostic significance of the criteria for CIMP-diagnosis established in the present study was clearly confirmed in the validation cohort.

Survival curves of the 100 patients belonging to the validation cohort were calculated by the Kaplan-Meier method (Figure 3). The period covered ranged from 27 to 5,031 days (mean: 1,860 days). Cancer-free (Figure 3A) and overall (Figure 3B) survival rates of patients with CIMP-positive ccRCCs diagnosed using the criteria based on the cutoff values listed in Table 1 were significantly lower than those of patients with CIMP-negative ccRCCs (*P* = 1.41 × 10^−5^ and 2.43 × 10^−13^, respectively, log-rank test).

Among 5 ccRCCs diagnosed as CIMP-positive in the validation cohort, one tumor was grade 2, two were grade 3, and two were grade 4 (Figure 2C); four were stage III and one was stage IV. Even after adjusting the grades, the cancer-free (*P* = 1.01 × 10^−3^) and overall (*P* = 7.04 × 10^−4^) survival rates of patients with CIMP-positive high-grade (grades 3 and 4) ccRCCs were significantly lower than those of patients with CIMP-negative high-grade (grades 3 and 4) ccRCCs (log-rank test, Additional file 9: Figure S3). The cancer-free (*P* = 7.76 × 10^−4^) and overall (*P* = 5.48 × 10^−5^) survival rates of patients with CIMP-positive high-stage (stages III and IV) ccRCCs were significantly lower than those of patients with CIMP-negative high-stage (stages III and IV) ccRCCs in the validation cohort (log-rank test, Additional file 9: Figure S3).

When compared with CIMP-negative ccRCCs, the CIMP-positive ccRCCs in the validation cohort had a significantly higher likelihood of recurrence (hazard ratio, 10.6; 95 percent confidence interval, 2.81 to 40.2; *P* = 5.03 × 10^−4^), and of disease-related death (hazard ratio, 75.8; 95 percent confidence interval, 7.81 to 735; *P* = 1.89 × 10^−4^) (Cox proportional hazards model). These data indicated that the validation cohort clearly demonstrated the prognostic significance of the criteria for CIMP diagnosis established in the present study.

## Discussion

Since the effectiveness of any therapy for relapsed or metastasized ccRCC is restricted unless it is diagnosed early by close follow-up after nephrectomy [3], significant prognostic criteria need to be established. Unlike alterations of mRNA and protein expression, which can be easily affected by the microenvironment of cancer cells, DNA methylation alterations are stably preserved on DNA double strands by covalent bonds [4, 5]. Therefore, DNA methylation levels at appropriate marker CpG sites would appear to be optimal prognostic indicators if evaluated quantitatively [27].

The present learning cohort comprised 88 CIMP-negative ccRCCs and 14 CIMP-positive ccRCCs: CIMP in the learning cohort was identified using hierarchical clustering based on single CpG-resolution Infinium assay in our previous study [7], which had revealed that CIMP-positive ccRCCs in the learning cohort were clinicopathologically aggressive tumors with a larger diameter, more frequent vascular involvement, infiltrating growth, and renal pelvis invasion, as well as having higher histological grades and pathological TNM stages than CIMP-negative ccRCCs [7] (Additional file 1: Table S1). During the follow-up period after nephrectomy, the cancer-free and overall survival rates of patients with CIMP-positive ccRCCs in the learning cohort were significantly lower than those of patients with CIMP-negative ccRCCs in our previous study [7], indicating that CIMP in ccRCCs might be applicable as a prognostic indicator.

We previously identified ccRCC-specific CIMP marker genes whose DNA methylation levels differed markedly between CIMP-negative and CIMP-positive ccRCCs based on the Infinium assay [7]. Since hierarchical clustering is not applicable to clinical use, in the present study we attempted to establish criteria for CIMP diagnosis that would be applicable to patients admitted to hospitals on an individual basis. The DNA methylation status of all promoter CpG islands, even CpG sites other than the Infinium probe sites, in the CIMP marker genes was evaluated quantitatively using the MassARRAY system, which is known to be suitable for quantification of multiple CpG sites [24]. Moreover, we carefully optimized the experimental conditions for MassARRAY analysis in order to avoid any PCR bias (Additional file 4: Table S4).

It was revealed that the entire promoter CpG islands in all the CIMP marker genes examined, i.e. *FAM150A, GRM6, ZNF540, ZFP42, ZNF154, RIMS4, PCDHAC1, KHDRBS2, ASCL2, PRAC, WNT3A, TRH, ZNF671* and *SLC13A5*, were methylated in CIMP-positive ccRCCs without exception (Figure 1 and Additional file 7: Table S5). Within such promoter CpG islands, there were many CpG sites where DNA methylation levels were useful for discrimination of CIMP-positive ccRCCs in the learning cohort from CIMP-negative ccRCCs (Additional file 8: Table S6). We identified the top 23 CpG units whose AUC values were larger than 0.95 in ROC analysis, and the Youden index was used as a cutoff value for such discrimination in each CpG unit (Table 1). The sensitivity and specificity of each of the 23 CpG units was sufficient for such discrimination (Table 1 and Figure 2A). Moreover, combination of the 23 CpG units generated criteria with 100% sensitivity and specificity for discrimination of CIMP-positive ccRCCs in the learning cohort from CIMP-negative ccRCCs (Figure 2B).

As a validation cohort, an additional 100 ccRCCs that had not been previously subjected to Infinium assay or hierarchical clustering were analyzed. The distribution of DNA methylation levels at the 23 CpG units in the validation cohort (Figure 2C) was quite similar to that in the learning cohort (Figure 2B), indicating that distinct DNA methylation profiles of the 23 CpG units are reproducible in ccRCCs. In the validation cohort, 5 ccRCCs were diagnosed as CIMP-positive based on the criteria established in the present MassARRAY analysis (Table 1). CIMP-positive ccRCCs diagnosed in the validation cohort had significantly lower cancer-free and overall survival rates than those of CIMP-negative ccRCCs (Figure 3). Even after adjusting the grades and stages, the cancer-free and overall survival rates of patients with high-grade (grade 3/4) and high-stage (stage III/IV) CIMP-positive ccRCCs were significantly lower than those of patents with high-grade (grade 3/4) and high-stage (stage III/IV) CIMP-negative ccRCCs (Additional file 9: Figure S3). Moreover, CIMP-positive ccRCCs had a higher likelihood of both recurrence and disease-related death (hazard ratios 10.6 and 75.8, respectively). These data indicated that CIMP of ccRCCs can be reproducibly diagnosed using the criteria established in the present study, and that CIMP diagnosis is useful for prognostication of patients with ccRCCs.

Reproducible diagnosis of CIMP using the criteria established in the present study makes it possible to explore the molecular background of CIMP-positive renal carcinogenesis. Since CIMP-positive ccRCCs show clinicopathological aggressiveness and poorer outcome [7], the molecular pathways participating in CIMP-positive renal carcinogenesis should be clarified and the therapeutic targets of CIMP-positive ccRCCs need to be identified. Even though we [28] and another group [29, 21] reported the results of multilayer omics analyses in ccRCCs, such reports did not focus on CIMP. Therefore we are now performing multilayer omics (i.e. genome (whole-exome), transcriptome and proteome) analyses of tissue specimens from CIMP-negative and -positive ccRCCs. Frequently affected molecular pathways that might potentially become therapeutic targets are now being identified in more aggressive CIMP-positive ccRCCs (unpublished data).

The criteria for CIMP diagnosis established in the present study may be useful for not only prognostication but also companion diagnostics for personalized medicine [30]. If our CIMP diagnosis reveals CIMP-negativity in samples of tumor tissue obtained by nephrectomy, the risk of recurrence and metastasis would be considered low, and such patients would not require adjuvant therapy. On the other hand, if our CIMP diagnosis reveals CIMP-positivity, then the risk of recurrence and metastasis would be considered high. Therefore, close follow-up and frequent imaging diagnosis are recommended for early diagnosis of recurrence. In addition, inhibitors for frequently affected molecular pathways identified by multilayer omics analysis in CIMP-positive ccRCCs might be effective after recurrence. If further preclinical examinations support the effectiveness of adjuvant therapy using inhibitors for frequently affected molecular pathways in CIMP-positive ccRCCs, such adjuvant therapy may be recommended immediately after nephrectomy in patients with CIMP-positive ccRCCs.

## Conclusions

CIMP of ccRCCs is characterized by accumulation of DNA methylation at CpG islands and poorer patient outcome. Based on quantification of DNA methylation levels of the ccRCC-specific CIMP marker genes, the criteria for CIMP diagnosis have been established. CIMP of ccRCCs can be reproducibly diagnosed using the criteria established in the present study. The prognostic significance of the criteria has been clearly validated in the validation cohort. Frequently affected molecular pathways that might potentially become therapeutic targets are now being identified using multilayer omics analyses in more aggressive CIMP-positive ccRCCs. The criteria for CIMP diagnosis may be useful for not only prognostication but also companion diagnostics for personalized medicine.

## Electronic supplementary material

Additional file 1: Table S1: Correlation between CpG island methylator phenotype (CIMP) and clinicopathological parameters of clear cell renal cell carcinomas (ccRCCs) in the learning cohort. (PDF 58 KB)

Additional file 2: Table S2: Clinicopathological characteristics of clear cell renal cell carcinomas (ccRCCs) in the validation cohort. (PDF 44 KB)

Additional file 3: Table S3: Microscopically examined tumor cell content (%) of specimens of clear cell renal cell carcinoma tissue from the learning and validation cohorts. (PDF 36 KB)

Additional file 4: Table S4: Primer sequences and optimal PCR conditions for MassARRAY. (PDF 7 MB)

Additional file 5: Figure S1: Standard curves for optimization of PCR conditions for the MassARRAY system on representative CpG units. To test the linearity of the protocol, 0%, 50% and 100% methylated control DNA was used as a template. Experiments were performed in triplicate for each sample-CpG unit, and the mean value for the three experiments was used as the DNA methylation level. Error bar: standard deviation. Optimized PCR conditions (annealing temperature and type of DNA polymerases) are summarized in Additional file 4: Table S4. (PDF 66 KB)

Additional file 6: Figure S2: Scattergrams of DNA methylation levels determined by Infinium assay and those determined by MassArray analysis. ^a^Probe ID for the Infinium HumanMethylation27 Bead Array. Six exact Infinium probe CpG sites (cg06274159 for the *ZFP42* gene, cg03975694 for the *ZNF540* gene, cg08668790 for the *ZNF154* gene, cg01009664 for the *TRH* gene, cg22040627 for the *SLC13A5* gene, and cg19246110 for the *ZNF671* gene) were examined by the MassArray platform in the learning cohort. Significant correlations between DNA methylation levels determined by our previous Infinium assay [7] and those determined by the present MassArray analysis were confirmed (*P* = 1.25 × 10^−35^, *P* = 1.98X10^−32^, *P* = 1.31 × 10^−41^, *P* = 5.30 × 10^−34^, *P* = 7.91 × 10^−22^ and *P* = 7.61 × 10^−44^, respectively). (PPTX 263 KB)

Additional file 7: Table S5: Differences of DNA methylation levels at all examined 193 CpG units including 299 CpG sites of 14 CpG island methylator phenotype (CIMP) marker genes between CIMP-negative and CIMP-positive clear cell renal cell carcinomas (ccRCCs) in the learning cohort. (PDF 75 KB)

Additional file 8: Table S6: Eighty-six CpG units showing area under the curve (AUC) values larger than 0.9 in receiver operating characteristic curve analysis for discrimination of CpG island methylator phenotype (CIMP)-positive clear cell renal cell carcinomas (ccRCCs) from CIMP-negative ccRCCs in the learning cohort. (PDF 40 KB)

Additional file 9: Figure S3: Kaplan–Meier survival curves of patients with CIMP-positive and -negative high-grade (grades 3 and 4) and high-stage (stages III and IV) clear cell renal cell carcinomas (ccRCCs) in the validation cohort. The cancer-free (Panel A, *P* = 1.01 × 10^−3^) and overall (Panel B, *P* = 7.04 × 10^−4^) survival rates of patients with CIMP-positive grade 3/4 ccRCCs were significantly lower than those of patients with CIMP-negative grade 3/4 ccRCCs (log-rank test). The cancer-free (Panel C, *P* = 7.76 × 10^−4^) and overall (Panel D, *P* = 5.48 × 10^−5^) survival rates of patients with CIMP-positive stage III/IV ccRCCs were significantly lower than those of patients with CIMP-negative stage III/V ccRCCs (log-rank test). Patients who underwent curative resection are included in panels A and C. (PDF 94 KB)

## Abbreviations

AUC: Area under the curve
CIMP: CpG island methylator phenotype
CV: Cutoff value
NCBI: National Center for Biotechnology Information
ccRCC: Clear cell renal cell carcinoma
ROC: Receiver operating characteristic.
