# Supplementary material for: Prognostication of patients with clear cell renal cell carcinomas based on quantification of DNA methylation levels of CpG island methylator phenotype marker genes
Source: BMC Cancer. 2014 Oct 20;14:772. doi: 10.1186/1471-2407-14-772 (PMC4216836; doi:10.1186/1471-2407-14-772)
Supplement: Supplementary file 1 — Additional file 1: Table S1: Correlation between CpG island methylator phenotype (CIMP) and clinicopathological parameters of clear cell renal cell carcinomas (ccRCCs) in the learning cohort. (PDF 58 KB) [file 12885_2014_4959_MOESM1_ESM.pdf]

Table S1. Correlation between CpG island methylator phenotype (CIMP) and clinicopathological parameters of clear cell renal cell carcinomas (ccRCCs) in the learning cohort.

Clinicopathological parameters		CIMP-negative ccRCCs (n=88)	CIMP-positive ccRCCs (n=14)	P^{a}
Age		62.32±10.21	67.36±11.06	9.90×10^{-2} [b]
Sex	Male	61	11	6.97×10^{-1} [c]
	Female	27	3	
Tumor diameter (cm)		4.96±3.02	8.75±2.85	$\underline{1.34\times10^{-4}}$ [b]
Histological grades[d]	G1	47	1	$\underline{7.69\times10^{-6}}$ [c]
	G2	33	4	
	G3	7	7	
	G4	1	2	
Vascular involvement[e]	Negative	52	1	$\underline{3.02\times10^{-4}}$ [c]
	Positive	36	13	
Renal vein tumor thrombi[f]	Negative	67	5	$\underline{2.05\times10^{-3}}$ [c]
	Positive	21	9	
Growth pattern	Expansive	82	7	$\underline{6.79\times10^{-6}}$ [c]
	Infiltrative	6	7	
Tumor necrosis	Negative	69	2	$\underline{1.26\times10^{-6}}$ [c]
	Positive	19	12	
Invasion to renal pelvis	Negative	81	10	$\underline{2.09\times10^{-2}}$ [c]
	Positive	7	4	
Pathological TNM stage[g]	Stage I	48	0	$\underline{7.25\times10^{-5}}$ [c]
	Stage II	1	1	
	Stage III	23	9	
	Stage IV	16	4	

Among the 104 RCCs used in our previous study [7], 102 (88 CIMP-negative and 14 CIMP-positive RCCs), from which sufficient amount of genomic DNA was available, were used in the present study as the learning cohort. [a]*P* values of <0.05 are underlined. [b]Mann-Whitney *U* test. [c]Fisher's exact test. [d]All the tumors were graded on the basis of previously described criteria [19]. [e]The presence or absence of vascular involvement was examined microscopically on slides stained with hematoxylin-eosin and elastica van Gieson. [f]The presence or absence of tumor thrombi in the main trunk of the renal vein was examined macroscopically. [g]All the patients were classified according to the pathological Tumor-Node-Metastasis (TNM) classification [20].
