# Supplementary material for: Prognostication of patients with clear cell renal cell carcinomas based on quantification of DNA methylation levels of CpG island methylator phenotype marker genes
Source: BMC Cancer. 2014 Oct 20;14:772. doi: 10.1186/1471-2407-14-772 (PMC4216836; doi:10.1186/1471-2407-14-772)
Supplement: Supplementary file 2 — Additional file 2: Table S2: Clinicopathological characteristics of clear cell renal cell carcinomas (ccRCCs) in the validation cohort. (PDF 44 KB) [file 12885_2014_4959_MOESM2_ESM.pdf]

Table S2. Clinicopathological characteristics of clear cell renal cell carcinomas (ccRCCs) in the validation cohort.

Clinicopathological parameters		Validation cohort (n=100)
Age		62.47±11.41
Sex	Male	68
	Female	32
Tumor diameter (cm)		5.67±3.05
Histological grades[a]	G1	40
	G2	40
	G3	15
	G4	5
Vascular involvement[b]	Negative	48
	Positive	52
Renal vein tumor thrombi[c]	Negative	72
	Positive	28
Growth pattern	Expansive	92
	Infiltrative	8
Tumor necrosis	Negative	79
	Positive	21
Invasion to renal pelvis	Negative	90
	Positive	10
Pathological TNM stage[d]	Stage I	52
	Stage II	11
	Stage III	26
	Stage IV	11

[a]All the tumors were graded on the basis of previously described criteria [19]. [b]The presence or absence of vascular involvement was examined microscopically on slides stained with hematoxylin-eosin and elastica van Gieson. [c]The presence or absence of tumor thrombi in the main trunk of the renal vein was examined macroscopically. [d]All the patients were classified according to the pathological Tumor-Node-Metastasis (TNM) classification [20].
