# Supplementary material for: Prognostication of patients with clear cell renal cell carcinomas based on quantification of DNA methylation levels of CpG island methylator phenotype marker genes
Source: BMC Cancer. 2014 Oct 20;14:772. doi: 10.1186/1471-2407-14-772 (PMC4216836; doi:10.1186/1471-2407-14-772)
Supplement: Supplementary file 3 — Additional file 3: Table S3: Microscopically examined tumor cell content (%) of specimens of clear cell renal cell carcinoma tissue from the learning and validation cohorts. (PDF 36 KB) [file 12885_2014_4959_MOESM3_ESM.pdf]

Table S3. Microscopically examined tumor cell content (%) of specimens of clear cell renal cell carcinoma tissue from the learning and validation cohorts.

Learning cohort

ID of tumors	Microscopically examined tumor cell content (%)
L1	57.5
L2	65.3
L3	69.2
L4	75.5
L5	49.1
L6	73.6
L7	66.2
L8	58.3
L9	90.0
L10	52.7
L11	50.7
L12	65.0
L13	59.1
L14	71.0
L15	60.4
L16	65.3
L17	65.6
L18	60.1
L19	76.4
L20	70.1
L21	66.0
L22	68.0
L23	79.2
L24	69.3
L25	75.0
L26	39.0
L27	47.4
L28	38.1
L29	58.5
L30	58.9
L31	49.4
L32	60.3
L33	63.0
L34	72.2
L35	68.3
L36	52.7
L37	77.1
L38	44.0
L39	73.4
L40	52.7
L41	67.9
L42	77.3
L43	50.4
L44	64.2
L45	65.7
L46	75.0
L47	60.5
L48	61.3
L49	40.7
L50	41.6
L51	61.4
L52	57.2
L53	72.8
L54	39.5
L55	45.6
L56	66.4
L57	51.8
L58	84.3
L59	39.4
L60	47.8

L61	52.8
L62	47.9
L63	57.6
L64	40.6
L65	74.0
L66	35.9
L67	37.8
L68	47.4
L69	51.8
L70	54.7
L71	56.5
L72	54.0
L73	41.8
L74	62.8
L75	95.8
L76	43.2
L77	44.6
L78	39.4
L79	40.1
L80	71.1
L81	47.1
L82	90.4
L83	76.2
L84	53.8
L85	35.8
L86	47.2
L87	71.7
L88	64.7
L89	47.3
L90	51.3
L91	77.3
L92	54.0
L93	62.2
L94	62.9
L95	55.2
L96	56.2
L97	46.1
L98	66.3
L99	76.2
L100	69.1
L101	92.5
L102	58.3

Validation cohort

ID of tumors	Microscopically examined tumor cell content (%)
V1	65.0
V2	69.2
V3	57.9
V4	61.0
V5	55.8
V6	67.5
V7	54.0
V8	70.8
V9	84.9
V10	58.3
V11	59.7
V12	81.6
V13	66.7
V14	51.6
V15	71.3
V16	88.1
V17	73.2
V18	63.4
V19	71.7
V20	62.8
V21	76.9

V22	59.3
V23	48.2
V24	73.9
V25	80.1
V26	79.0
V27	62.6
V28	57.6
V29	64.3
V30	74.7
V31	60.6
V32	62.0
V33	38.0
V34	59.8
V35	76.6
V36	60.0
V37	44.7
V38	68.3
V39	58.9
V40	81.0
V41	71.7
V42	47.2
V43	47.2
V44	70.4
V45	95.5
V46	68.7
V47	48.3
V48	53.3
V49	41.3
V50	80.0
V51	82.8
V52	67.1
V53	70.3
V54	30.2
V55	59.5
V56	64.8
V57	74.0
V58	77.3
V59	68.1
V60	68.4
V61	63.5
V62	69.5
V63	55.4
V64	73.1
V65	70.5
V66	84.4
V67	56.5
V68	62.6
V69	46.4
V70	66.3
V71	66.5
V72	53.2
V73	47.4
V74	51.7
V75	79.0
V76	60.6
V77	82.4
V78	66.3
V79	68.8
V80	61.1
V81	73.9
V82	72.0
V83	54.7
V84	80.3
V85	61.4
V86	62.3
V87	66.3
V88	81.4

V89	68.8
V90	57.6
V91	74.2
V92	58.8
V93	46.6
V94	83.3
V95	70.2
V96	81.8
V97	64.2
V98	84.5
V99	53.4
V100	75.4
