# Supplementary material for: Prognostication of patients with clear cell renal cell carcinomas based on quantification of DNA methylation levels of CpG island methylator phenotype marker genes
Source: BMC Cancer. 2014 Oct 20;14:772. doi: 10.1186/1471-2407-14-772 (PMC4216836; doi:10.1186/1471-2407-14-772)
Supplement: Supplementary file 4 — Additional file 4: Table S4: Primer sequences and optimal PCR conditions for MassARRAY. (PDF 7 MB) [file 12885_2014_4959_MOESM4_ESM.pdf]

Table S4. Primer sequences and optimal PCR conditions for MassARRAY.

Gene symbol	ID of CpG units	Size of amplicon (bp)	Primer sequences		Target Sequence (Examined CpG units are marked in yellow. Examined CpG sites in each CpG unit are underlined. CpG sites which were present, but could not be evaluated by MassARRAY, are indicated by dotted underlining.)	Optimized PCR condition	
			Forward primer	Reverse primer		Annealing temperature (ºC)	Polymerase
FAM150A	1-17	455	aggaagag<br>agGGGA<br>GGATTTA<br>GTAGGG<br>TAATTGT	cagtaatac<br>gactcactat<br>agggagaa<br>ggctTTTC<br>ACCTAAA<br>AAAACAC<br>TAAAACC	GGGAGGACCCAGTAGGGTAACTGCCGCGTCGCCCCGGCGGTTCTCCCTGGGC<br>TCTGTCTCCCGCCGCCTCCACCCCCCGAGCCTCGGGGTCCGTCACGGCTTCC<br>CCTGGCTGGCGGGGTCAGTAGAACCCGCGGCGCCTAGGTCCGGACGGAAAAA<br>AGCAGGGCCGGGGTGCGGCCTGGATGAGCGGAGATCTCCGCGCCTTGGGCTC<br>AAAGGTGCGGGGTGCGCTCTGCTGCCGAGCCCCTGCTCGCTCAGGAACACTG<br>GCCACGCCGTCACGCCAGCCGCCCCTGCCCCAGGTCTGGAGGCCCGACCTGC<br>TCTCCTAGGCGCAGCACCGCGTTCTCTTCCGCGTGGGGGAGCGGCGGGCGGA<br>AGAGGTCTGGGGCTGGGCACCGGGGACACGCGCCCAGCTCCCCTGGCCTCCC<br>TGGGGGGAGTGGCCGGTTTCAGTGCTTCCCCAGGTGAAA	60	TaKaRa Taq HS DNA polymerase (Takara Bio, Shiga, Japan)
GRM6	18,19	188	aggaagag<br>agGGTTT<br>AGGATAA<br>GTTTGTG<br>ATAGATG	cagtaatac<br>gactcactat<br>agggagaa<br>ggctAAAA<br>CAAAAAA<br>ACAAACC<br>CAAAAAT	GGTTCAGGACAAGTCTGTGACAGATGCGGCCGAGGCCCTGAGCGAGAGAGGA<br>TTTAAGGATTCTAGGGAGGGATGAGAGACCGCTCCGAGGGTGGAGACCCCTCC<br>TGAGTGTGGGGGGTGGCGGTGCTGGCTCTCCCCGCATCTCCTTCCCCTCCCTC<br>TCCCAATCTCTGGGTCTGTTTTCCTGTTTT	56	HotStar Taq DNA polymerase (QIAGEN GmbH, Hilden, Germany)
ZFP42	20-25	196	aggaagag<br>agGAGTT<br>GATGGG<br>TGGTTGT<br>AGTTT	cagtaatac<br>gactcactat<br>agggagaa<br>ggctCCCA<br>TTTAAAA<br>AAAATTC<br>CATAAAA<br>CAAA	GAGCTGATGGGTGGCTGTAGCCTGATTAGACCGCGTCAGTCCGGAGGGTGGG<br>TCTTGGGAGGGGGCGCAGGGCAGTCCACGTTTCCACTGCAGTTTCTCCTTTGT<br>TTTACGTTTGGGAGGAGGTGGCATTGGAAATAGCAGAGTGCTTCGCGGTAACA<br>GGGGTGAGTCTTGTTTCATGGAACTTTTTTCAAATGGG	56	HotStar Taq DNA polymerase (QIAGEN)

ZNF540	26-42	463	aggaagag<br>agGGGTA<br>GGGTAG<br>AATTAGG<br>TTAAAGA<br>AA	cagtaatac<br>gactcactat<br>agggagaa<br>ggctACTA<br>AAATCAA<br>TAACCCC<br>CAAAAAA	GGGCAGGGCAGAACCAGGCTAAAGAAACGTCCAGCGTAGCTTCAAGGATGCAC<br>CGCGTGATCCCTATCGGATCTCCCCGACGCGTCAGGCCTGCCTAGACGGTGCT<br>GGGAGCGCGTCTCCTTGAACGTTGTCCCGCCTGGGATTGCGAGGTAGGTACC<br>GCCTGCCTGTGTGTACCGGGGCTGCTGTCTCCGGGGAGGGGCTTCTGGCGGA<br>CAGGAGAACCAAGCAGCCTCAGGAGCTGCCTGGGTGTGTGTGTTTCTGTGCGA<br>GTGTTGCATATCTCTGTGTGTGTTTCTGTGCAAGTGTTGCATGTCTGTGTGTGT<br>GGGGGGGGTGGTGTCTGGTGAAAAGAATGTGTCTCGTGCGGTGGAGCGCCGT<br>TTCTCTGTAGTCCGCGGGCTCTCTTATGCGCCCTCTTGTGGTCCCAAGTGTGCT<br>TTTCTTGTTTTTCGCTTTCTTGGGGGTCATTGATTTCAGC	58	TaKaRa Taq<br>HS DNA<br>polymerase<br>(Takara)
ZNF154	43-48	279	aggaagag<br>agGGTGA<br>ATATATT<br>TTAGAGA<br>AGTTAAA<br>ATGG	cagtaatac<br>gactcactat<br>agggagaa<br>ggctTCCC<br>TCCACTA<br>CCCTAAA<br>ACTTAAA	GGTGAACACACCTCAGAGAAGCTAAAATGGCCGCCACGAAGAGGCCCCCCCAA<br>AAGTCCCGTCCTTTCTTTTTGTGACTCTCAAGGAAAGTCGGTTTTCTGAGCTCTT<br>ACTGGCTTAGTAGCGTGGCGTTCAACGCAGAGCATTCTAGGTAATGTAGTTTTC<br>ATAGATCCCGAGGTGGGTGCCGGGGACCCTTTGCACCAACCTCTTGGAGTAAA<br>AGCGAAGCTCCAGGGCGCTGGGCGATGAGAAATGGCTTATCCAAGTCCTAGGG<br>CAGTGGAGGGA	54	HotStar Taq<br>DNA<br>polymerase<br>(QIAGEN)
RISM4	49-59	404	aggaagag<br>agGGAGT<br>TTTAGTT<br>TATGAG<br>GGAAGG<br>A	cagtaatac<br>gactcactat<br>agggagaa<br>ggctAAAC<br>CCCAAAA<br>TCTCCAA<br>AATAC	GGAGCCCCAGCCCATGAGGGAAGGAGAGGAGAGATAAATGGGGGCGCTCAAG<br>GCCTGGGGCGCCGGGCAGGGGTCTTGGGCAGGGATCCTCTGGATGTGGCCAA<br>GACAAAGATGGAGAGGTAAGGTCTGCGCGCCACCTCCAATGGCGGGGGGCGC<br>GTCGGAGCCCCAGGGGTGGGACGGCCAAAGCCCAGGGCTTGAAGAGTGGGC<br>ACATTCAGGAGACTCAGGGAGGGTGGCAGGTCGGCTCCAGGGACGAGGCAAG<br>GGGCCTCCAATAGGCGCGGGTGAGGAGGGAGATGGGTCCTGGCGACCCAAAG<br>GGCCCACCTGCGGGAAAGGTGAATGCAGACAATCTCGGGGTCCCTGGGGGAG<br>AAGGCCAGAAGGTAGCGCATCCTGGAGACCCTGGGGTCC	54	HotStar Taq<br>DNA<br>polymerase<br>(QIAGEN)
PCDHAC1	60-73	362	aggaagag<br>agTGGTA<br>GTTTTTG<br>GGATATA<br>AGAGGG	cagtaatac<br>gactcactat<br>agggagaa<br>ggctAAAC<br>TACCCAA<br>ATCTTAA<br>CCTCCA<br>C	TGGCAGCTCCTGGGATACAAGAGGGTGCAGGACAGACTTCAACCCGCAGCAG<br>GATCCAGCGCGGAAAGCTCTGCAGCAGGATCCAGCGCGGAAAGCCCCCCGCA<br>GCACTTCTTTCGGGGGGCTCCTGTTTCCTTAAGCCTAGAAGGTGTGGTCGCTCA<br>CGTTCACCGTCCCGCCTCTCGCCGCCTCCGCTCGGCAGCTCCACGCTGAGTCC<br>CGCCCTCTCCGCCGGAGAGGTGCGCCGGGGTCAGAGCGCCGGGACCCGACG<br>CGCGGCTCCCAAAGGGCGGCAGGAAGAGCCCAGCTGGGCTCAGCCACAGTTA<br>TCAGCAATCTGCGGGCAGAGGATGTGGAGGTTAAGATCTGGGCAGCCT	56	HotStar Taq<br>DNA<br>polymerase<br>(QIAGEN)

PRAC	74-78	264	aggaagag<br>agGGTGA<br>AAGTTTG<br>TTGTTTA<br>TTTTTTT<br>T	cagtaatac<br>gactcactat<br>agggagaa<br>ggctCAAA<br>CTAAATT<br>CTAATCC<br>CCACCTT	GGTGAAAGCCTGCTGCTCACCCTTCCCTTGTTTCCCAAAACTTCTGAAGGCTCC<br>CAAATTCCTGGGAGACCCTCTCCCAGGGCCTCCTGATGCAGCTACCATACTGA<br>GCGATCCGTCGATAACGCCCTTGGCCCACCGATCAGTTTACCTTATTAGAGAGA<br>AAAGCACTCTTGGAGGTAGTAAGATGGGCCGGTCCTTGATCTGAGAAATGGGC<br>GCACAACATCGCTGTTCTCTCTGCAAAGGTGGGGACCAGAATCCAGCTTG	58	HotStar Taq<br>DNA<br>polymerase<br>(QIAGEN)
TRH	79-97	414	aggaagag<br>agAATAG<br>ATTTTTA<br>GAGGTG<br>GTGTAG<br>AAA	cagtaatac<br>gactcactat<br>agggagaa<br>ggctAAAA<br>AACTCCC<br>TTTCCAA<br>TACTCC	AACAGATCTCCAGAGGTGGTGCAGAAACGACCCCGCGCCGGCGCCCCATCCT<br>GCGGCCAGTGCCTCCGCGCCCCGGCTCCGGTCCCCACCGTCCCCGCCCCAGA<br>TTTCCGGAGGAGCAGGCGGGCGGGGTCCCGCGGGGCCGGCTGCCGTCAGCG<br>CCCCTTCCCGGCGGCCGCGACCCCTCCCCGCTGACCTCACTCGAGCCGCCGC<br>CTGGCGCAGATATAAGCGGCGGCCCATCTGAAGAGGGCTCGGCAGGCGCCCG<br>GGGTCCTCAGCGCTGCAGACTCCTGACCTGCCGACTGCGGATCCCGAGTCCC<br>CGGATCCCGGACCCATCCTGTGGAGCCCACTCCTGGCAGGTAACCGCCCCAA<br>CCCCTCTCCTTCCGCAGACGGTGTCCGGGAGCACTGGAAAGGGAGCCCTCT	54	HotStar Taq<br>DNA<br>polymerase<br>(QIAGEN)
SLC13A5	98-114	500	aggaagag<br>agGAAGG<br>ATTTGAA<br>TTTGGAG<br>ATATAGT<br>TT	cagtaatac<br>gactcactat<br>agggagaa<br>ggctAAAA<br>AACCCAA<br>AAACCTA<br>CAAAAAA	GAAGGACTTGAACTTGGAGACATAGCTCAGCGCCGAGGCCATCGCGCGGGAG<br>GGAGACTGGCGGGCGAGACGAGTGAGGGGCAGCTAGAGGCGCCGCGGGCTT<br>AAGAAGGGGCCACAGTCCCCGGGGATTGGGGAGGGGGCGGTGACAACTCCG<br>CCCCGCACGGGGGCGCCTCCCCGCGGCCCTGGGGCGGGGCCACCCCTCGGG<br>GTCTGTGGGACGCGCCTGCCCCCAATTCTGCCACCCGGCGGCGGTGGGAGGC<br>GTCTTTGGACTCCAACGCTTCGGGCCAGCCCTCTAGGGGCAGCCTGGGCCCTA<br>GCATCTCGCGCTGTCCAAGCCTCTCCTGCGCTGCCGAGGCAGAGGTGCGTCC<br>CGGGGCTGCCAAGCGGGGCGTGTTTTGGTCACTGGTGCTGCCCGCTTTGGCG<br>TAAGGCGCCCTCCCGCGTCCGCATCTGCTCTTTCCTGGGCTCTGAAGGGTCCC<br>GGATGAAACTCTCTGCAGGCCTCTGGGTCTCTC	54	HotStar Taq<br>DNA<br>polymerase<br>(QIAGEN)
SLC13A5	115-130	463	aggaagag<br>agTTTTTT<br>TGGGTTT<br>TGAAGG<br>GTT	cagtaatac<br>gactcactat<br>agggagaa<br>ggctTTAT<br>ATCCCTT<br>CCTCTCT<br>AAAACTC<br>C	CTTTCCTGGGCTCTGAAGGGTCCCGGATGAAACTCTCTGCAGGCCTCTGGGTC<br>TCTCAGGTCTATCTCCCCGATCTCCCTCTCCTTTCCATCTCCTTACTTCCGCCCC<br>TCCGGTGTCTCTCCGAGAGGTGTCCCCCCACGCCCCGCGCCCTCCGCACCGC<br>GGGCCTCGCTTCCCGGTCCCCCCTGGCTTCCTCGCCACGTCCGCCCCACTCTA<br>GGTGCAGGACCCCTTTTCCCCGCTCGCACTCTCCGGCCCCGGAGCTCCTGGGC<br>GATCGCACAGGGAAGCGAGGCCACTGTCCTCCTCTGTCCCAGGGGCTGTCGC<br>GCTCCAGTGGACGCTGCACCCCGCAGACGCCCCGGCGGGCAGATGCGGACAC<br>GCGTCTTGGAGGGGCCCCACCGAGCCTCAGCAGCCGCAGCTGCCCGCCCGAC<br>CCAGGTCAGAGGGAAACGGAGCTCTAGAGAGGAAGGGACACAA	58	HotStar Taq<br>DNA<br>polymerase<br>(QIAGEN)

SLC13A5	123-135	384	aggaagag<br>agTTTTTT<br>TTGTTTT<br>AGGGGT<br>TGT	cagtaatac<br>gactcactat<br>agggagaa<br>ggctCCAC<br>CAACATA<br>AATAAAA<br>CTCCCC	CCTCCTCTGTCCCAGGGGCTGTCGCGCTCCAGTGGACGCTGCACCCCGCAGA<br>CGCCCGGCGGGCAGATGCGGACACGCGTCTTGGAGGGGCCCCACCGAGCCT<br>CAGCAGCCGCAGCTGCCCGCCCCGACCCAGGTCAGAGGGAAACGGAGCTCTAG<br>AGAGGAAGGGACACAACTAAGGCGACACTGAGACAGTCGCCCATGTATTCATT<br>CAGCCCGCCAGGCAACAGACAGGTGCCGAGCACCTCTTCTCCGCGAGGCCCT<br>GTTTTGGGCACTGGAGACACACGGATGCAAAGACATCCCCACCTCTGTGATTTT<br>CTTCTTTCCTCTCCTCTGCCTGCCTCTCATTCTGCAGCTTCCTTTTGGGGAGTCT<br>CATCCATGCTGGTGG	58	HotStar Taq<br>DNA<br>polymerase<br>(QIAGEN)
ZNF671	136-156	428	aggaagag<br>agTGGGA<br>TATAGG<br>GGTTGTA<br>GGTATTT	cagtaatac<br>gactcactat<br>agggagaa<br>ggctATAA<br>AAACCAC<br>ACTCTAC<br>CCACAAA	TGGGACACAGGGGCTGCAGGCACTTTACGATTCGGAGTCGGAGAAAGGGTGA<br>CTGAGGGCCCGGAGGACGCAGCACCCACCCGCGCGGAGTCCGTTAGCTCCGC<br>CATAGGACCGTGGGCGCGGACAGCTGCCGGGAGCGGCAGGCGTCTCGATCG<br>GGGACGCAGGCACTTCCGTCCCTGCAGAGCATCAGACGCGTCTCGGGACACT<br>GGGGACAACATCTCCTCCGCGCTTTCCCAACACCTCCACCTGCGGCCCACACA<br>AGCGTTACAGAACCCCGGCCAGGGACAGCCTGACAGAAACAAAATGTCCGCTA<br>CAAGGAGGAGCCGGAAGTCCCGCCCACGCACCCCCCGCAGGCACTGAAACAC<br>CCCTCTCCTGGGCCCTCATTGGGTATGCAACGTATAGGTTTGTGGGTAGAGTGT<br>GGTTCCCAC	58	HotStar Taq<br>DNA<br>polymerase<br>(QIAGEN)
WNT3A	157-165	348	aggaagag<br>agGTTTA<br>TTTGGTA<br>ATGAGG<br>GGTTGTT	cagtaatac<br>gactcactat<br>agggagaa<br>ggctTTCC<br>TCAATCT<br>TAAACAT<br>CTCAAAA	GTCCACCTGGCAATGAGGGGCTGCTGTAGAGAGAGTTAAGGGTGAGTTAAGCA<br>CGGGGTGTGAGGGGCTCCAGGACCCTCAATCAGAAAGCGCTGTGCTGCGCCC<br>TCCACACCAGAAAAGGCGCGTTCCGTGAGACCCTCCCCAGCCTGGCGATGGAA<br>GTGCAGATAAACCAAAGGAAGGGTCCCGAAAGGTCTCTCTCAGGCCTCCCACC<br>TCCACTGCACATATCCTGTGGGAGGGGGAACGGTGGCCACACTTTCGCCAGGG<br>CTTGTGATCCCTCAGAGCCCTCACCAAGCAAGGATCACCCCAGTTCCGAATTAA<br>GGGCGCTCTGAGATGCCCAAGATTGAGGAA	58	HotStar Taq<br>DNA<br>polymerase<br>(QIAGEN)
KHDRBS2	166-181	422	aggaagag<br>agTTTGG<br>TATTATT<br>ATTAATG<br>AGTGGTT<br>GG	cagtaatac<br>gactcactat<br>agggagaa<br>ggctAACA<br>AATCCTA<br>CCTTCTA<br>CCAAAAA<br>A	CCTGGCACCACCACCAATGAGTGGTTGGCGGAGGTGGGCCGCGTCCTGTTCC<br>CGCCTCTCCAGTTAAGGCCGCTGGTGTGAGCCGGGGCTCTGCGCGAGCGAGG<br>GACGACGGAAGGGACGGGCAGGTGTGGGCGCGGGGCCACGCAGCCCGACGG<br>CGGGAGTCGCAGGTGCTGGGTGCATGGGCCAGTGAGGACGCACAGAGATCCC<br>TCGCCGCGCGGAGGAGGAGCAGCGCGGGAGCCAGGCGCTGCCCCAAGACCC<br>TGCCTGCGTCCGAGCGAGCGGAACCTCGCGCTTCGCCCGGGGACAATCCGAA<br>GTCCGCGCTATGGAAGAGGAGAAATATTTGCCTGAGCTGATGGCAGAGAAAGA<br>TAGCCTGGATCCATCTTTTGTGCATGCGTCGCGCCTTTTGGCAGAAGGTAGGAC<br>TTGCC	56	HotStar Taq<br>DNA<br>polymerase<br>(QIAGEN)
ASCL2	182-193	339	aggaagag<br>agGTTAA<br>TAAAGTT<br>GGGTTTT<br>TGTTGG	cagtaatac<br>gactcactat<br>agggagaa<br>ggctAATA<br>CAAACCT<br>CCAAAC<br>CCTCC	GCTAACAAAGCTGGGTTCCTGCTGGGCCCCGCCCTGCTCCTCGCCCCCGCGA<br>CTGGGCTGGGCGCGCTGTCCCCTAGCGCAGCTATGTCCCGAGCGCGCCCCCA<br>CCTGTGCGTTAATCTACTGGGAATGGGGGTGGACTGCGCCTTACCTGGGGCGG<br>GGTGGGGCTTAAGGAGTGGTCGAGACTGAGGCGGGGTGGGAGGTTCAGGTTC<br>CCGGGGCGCCTTCCCCAACCCGCCCCGCTTTCCCCGTCCCTCCACGCGCACC<br>CTGCCTGTGGTTTCCGTGCGCCCCCGGCCTGAGGGCTCTGGGCGGCACCTTA<br>ACCCGGAGGGCCTGGAGGTCTGCACC	58	HotStar Taq<br>DNA<br>polymerase<br>(QIAGEN)
