# Supplementary material for: Prognostication of patients with clear cell renal cell carcinomas based on quantification of DNA methylation levels of CpG island methylator phenotype marker genes
Source: BMC Cancer. 2014 Oct 20;14:772. doi: 10.1186/1471-2407-14-772 (PMC4216836; doi:10.1186/1471-2407-14-772)
Supplement: Supplementary file 5 — Additional file 5: Figure S1: Standard curves for optimization of PCR conditions for the MassARRAY system on representative CpG units. To test the linearity of the protocol, 0%, 50% and 100% methylated control DNA was used as a template. Experiments were performed in triplicate for each sample-CpG unit, and the mean value for the three experiments was used as the DNA methylation level. Error bar: standard deviation. Optimized PCR conditions (annealing temperature and type of DNA polymerases) are summarized in Additional file 4: Table S4. (PDF 66 KB) [file 12885_2014_4959_MOESM5_ESM.pdf]

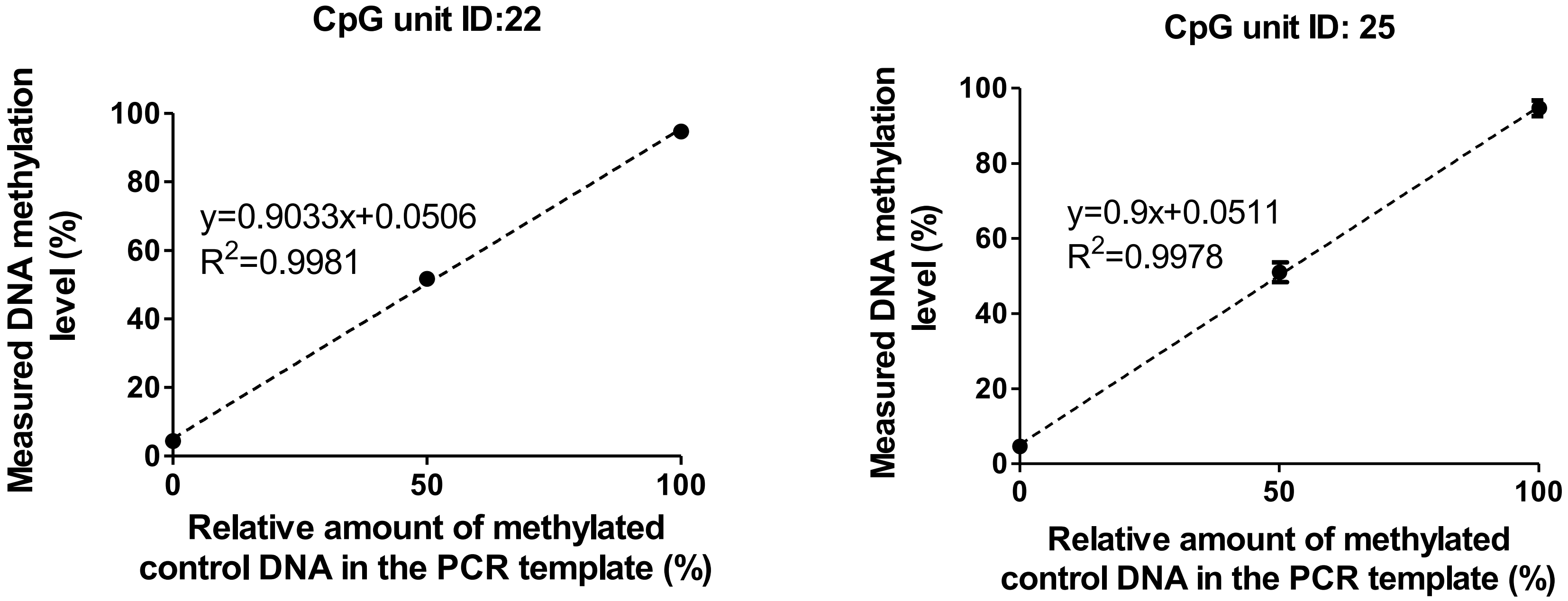

Figure S1. Standard curves for optimization of PCR conditions for the MassARRAY system on representative CpG units. To test the linearity of the protocol, 0%, 50% and 100% methylated control DNA was used as a template. Experiments were performed in triplicate for each sample-CpG unit, and the mean value for the three experiments was used as the DNA methylation level. Error bar: standard deviation. Optimized PCR conditions (annealing temperature and type of DNA polymerases) are summarized in Additional file 4: Table S4.
