# Supplementary material for: Prognostication of patients with clear cell renal cell carcinomas based on quantification of DNA methylation levels of CpG island methylator phenotype marker genes
Source: BMC Cancer. 2014 Oct 20;14:772. doi: 10.1186/1471-2407-14-772 (PMC4216836; doi:10.1186/1471-2407-14-772)
Supplement: Supplementary file 6 — Additional file 6: Figure S2: Scattergrams of DNA methylation levels determined by Infinium assay and those determined by MassArray analysis. aProbe ID for the Infinium HumanMethylation27 Bead Array. Six exact Infinium probe CpG sites (cg06274159 for the ZFP42 gene, cg03975694 for the ZNF540 gene, cg08668790 for the ZNF154 gene, cg01009664 for the TRH gene, cg22040627 for the SLC13A5 gene, and cg19246110 for the ZNF671 gene) were examined by the MassArray platform in the learning cohort. Significant correlations between DNA methylation levels determined by our previous Infinium assay [7] and those determined by the present MassArray analysis were confirmed (P = 1.25 × 10^−35^, P = 1.98X10−32, P = 1.31 × 10^−41^, P = 5.30 × 10^−34^, P = 7.91 × 10^−22^ and P = 7.61 × 10^−44^, respectively). (PPTX 263 KB) [file 12885_2014_4959_MOESM6_ESM.pptx]

## Slide 1
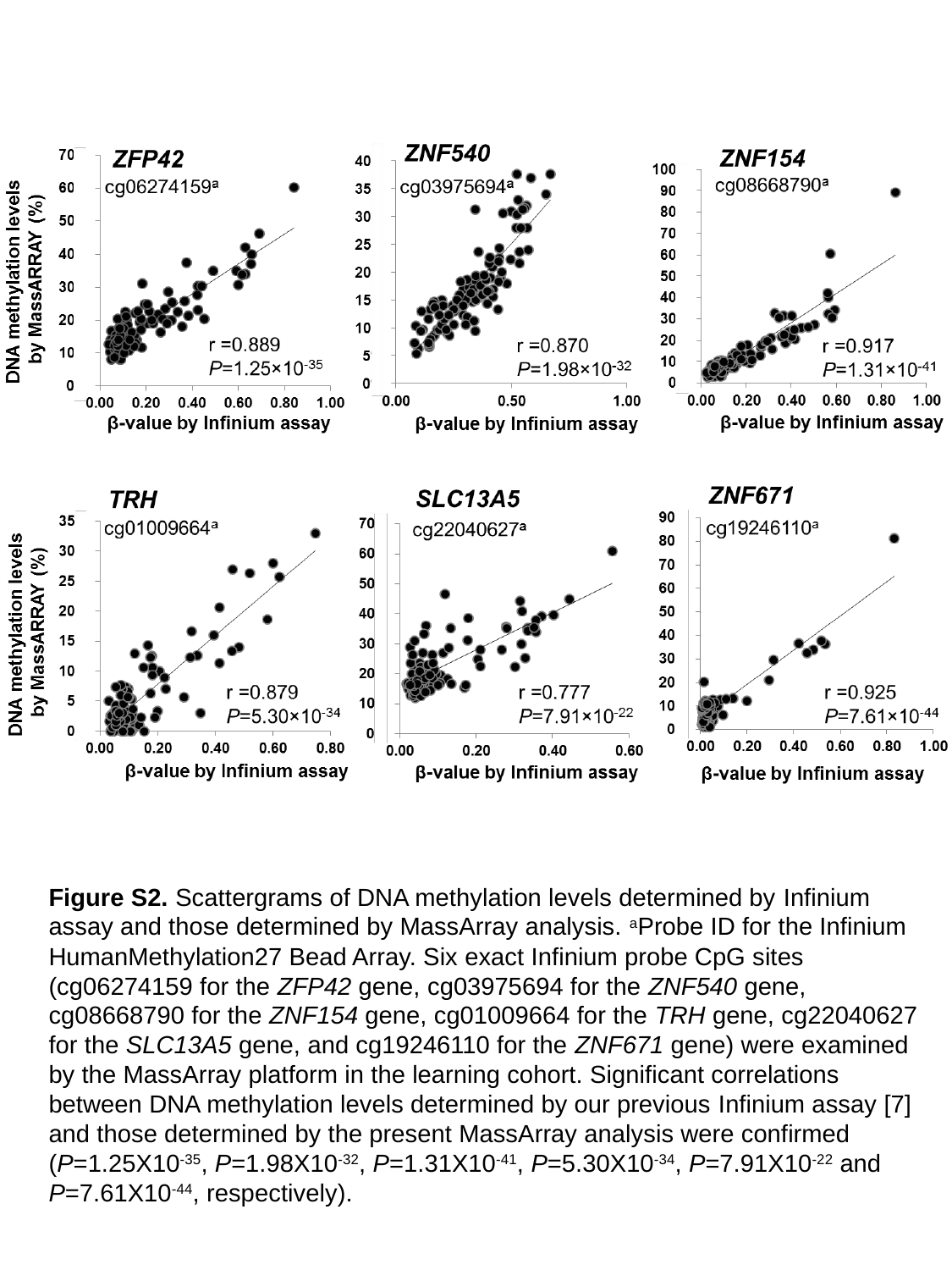

Figure S2. Scattergrams of DNA methylation levels determined by Infinium assay and those determined by MassArray analysis. aProbe ID for the Infinium HumanMethylation27 Bead Array. Six exact Infinium probe CpG sites (cg06274159 for the ZFP42 gene, cg03975694 for the ZNF540 gene, cg08668790 for the ZNF154 gene, cg01009664 for the TRH gene, cg22040627 for the SLC13A5 gene, and cg19246110 for the ZNF671 gene) were examined by the MassArray platform in the learning cohort. Significant correlations between DNA methylation levels determined by our previous Infinium assay [7] and those determined by the present MassArray analysis were confirmed (P=1.25X10-35, P=1.98X10-32, P=1.31X10-41, P=5.30X10-34, P=7.91X10-22 and P=7.61X10-44, respectively).
