# Supplementary material for: Prognostication of patients with clear cell renal cell carcinomas based on quantification of DNA methylation levels of CpG island methylator phenotype marker genes
Source: BMC Cancer. 2014 Oct 20;14:772. doi: 10.1186/1471-2407-14-772 (PMC4216836; doi:10.1186/1471-2407-14-772)
Supplement: Supplementary file 7 — Additional file 7: Table S5: Differences of DNA methylation levels at all examined 193 CpG units including 299 CpG sites of 14 CpG island methylator phenotype (CIMP) marker genes between CIMP-negative and CIMP-positive clear cell renal cell carcinomas (ccRCCs) in the learning cohort. (PDF 75 KB) [file 12885_2014_4959_MOESM7_ESM.pdf]

Table S5. Differences of DNA methylation levels at all examined 193 CpG units including 299 CpG sites of 14 CpG island methylator phenotype (CIMP) marker genes between CIMP-negative and CIMP-positive clear cell renal cell carcinomas (ccRCCs) in the learning cohort.

ID of CpG unit	Gene symbol	Chromosome	Position of CpG site [a]	DNA methylation level (%) (mean±SE)		*P* value [b]
				CIMP-negative ccRCCs (n=88)	CIMP-positive ccRCCs (n=14)	
1	FAM150A	8	58478309	7.10±0.30	16.90±3.40	1.81E-05
2	FAM150A	8	58478316, 58478323	5.30±0.30	18.00±4.60	7.71E-08
3	FAM150A	8	58478361, 58478363, 58478366	8.20±0.40	22.90±4.20	1.08E-06
4	FAM150A	8	58478376	5.30±0.50	15.30±2.70	2.07E-06
5	FAM150A	8	58478396, 58478403	13.70±0.50	28.80±3.60	1.83E-07
6	FAM150A	8	58478416	41.60±2.50	54.40±7.10	3.61E-02
7	FAM150A	8	58478426, 58478428	25.30±0.80	45.20±3.90	2.34E-07
8	FAM150A	8	58478477	17.70±0.70	37.60±3.10	6.20E-08
9	FAM150A	8	58478496, 58478499	16.60±0.60	37.70±3.80	2.21E-07
10	FAM150A	8	58478504	2.20±0.40	16.50±3.60	9.96E-07
11	FAM150A	8	58478511	17.70±0.70	37.60±3.10	6.20E-08
12	FAM150A	8	58478536	3.30±0.40	17.90±4.70	4.05E-05
13	FAM150A	8	58478553	10.20±0.80	33.20±4.90	1.41E-05
14	FAM150A	8	58478585, 58478588, 58478592	13.20±0.80	31.80±4.20	1.15E-06
15	FAM150A	8	58478616	13.90±1.30	41.00±5.10	8.56E-06
16	FAM150A	8	58478624, 58478626	16.60±0.60	37.70±3.80	2.21E-07
17	FAM150A	8	58478661	8.30±0.50	23.40±4.30	5.02E-06
18	GRM6	5	178422320, 178422324	10.10±0.77	32.04±3.91	1.37E-06
19	GRM6	5	178422375, 178422380	8.63±0.37	22.47±2.74	2.29E-07
20	ZFP42	4	188916865, 188916867	17.00±0.67	32.55±3.30	8.77E-06
21	ZFP42	4	188916875	10.23±0.61	25.67±2.90	5.73E-07
22	ZFP42	4	188916899	9.80±0.55	26.83±3.83	2.03E-07
23	ZFP42	4	188916913	9.33±0.39	24.31±3.27	2.85E-07
24	ZFP42	4	188916943	25.67±1.20	46.69±3.90	3.41E-06
25	ZFP42	4	188916982, 188916984	20.67±0.98	42.19±2.99	2.28E-07
26	ZNF540	19	38042446	46.15±1.15	60.42±3.90	1.00E-04
27	ZNF540	19	38042453	30.51±1.66	50.92±5.17	6.64E-01
28	ZNF540	19	38042472, 38042474	15.26±0.77	27.95±2.52	3.03E-08
29	ZNF540	19	38042486	30.51±1.66	50.92±5.17	2.11E-03
30	ZNF540	19	38042496	23.81±1.53	48.80±4.08	3.22E-08
31	ZNF540	19	38042499, 38042501	17.65±1.21	36.03±4.88	1.92E-06
32	ZNF540	19	38042518	20.25±1.57	44.45±2.75	2.12E-07
33	ZNF540	19	38042530, 38042532	21.48±1.17	45.40±2.10	7.60E-08
34	ZNF540	19	38042544, 38042552	40.92±1.09	55.75±2.86	1.47E-05
35	ZNF540	19	38042564	17.67±1.49	38.33±6.96	1.16E-03
36	ZNF540	19	38042576	22.31±0.88	35.92±3.35	3.46E-07
37	ZNF540	19	38042593, 38042608	40.68±1.65	51.10±5.51	1.24E-02
38	ZNF540	19	38042625	24.31±1.57	41.88±7.96	1.64E-05
39	ZNF540	19	38042770, 38042774	23.65±1.33	40.90±4.46	4.83E-07
40	ZNF540	19	38042782, 38042785	21.63±1.16	38.57±4.79	2.96E-06
41	ZNF540	19	38042800, 38042802	35.12±1.26	57.15±4.62	1.89E-08
42	ZNF540	19	38042816	26.95±1.18	48.07±5.15	8.69E-05
43	ZNF154	19	58220567	7.61±0.59	30.67±5.37	4.55E-08
44	ZNF154	19	58220627	5.75±0.54	32.93±5.47	2.30E-08
45	ZNF154	19	58220657, 58220662	10.37±0.69	35.71±5.08	3.67E-08
46	ZNF154	19	58220706	10.67±0.67	26.02±5.26	8.10E-07
47	ZNF154	19	58220718	14.70±1.10	31.07±5.40	9.78E-05
48	ZNF154	19	58220766, 58220773	26.58±1.31	50.31±3.96	5.76E-07
49	RIMS4	20	43438319	11.11±1.05	20.86±3.40	1.26E-03
50	RIMS4	20	43438335, 43438338	16.56±0.84	28.42±3.96	2.80E-04
51	RIMS4	20	43438404, 43438406	30.49±1.47	52.19±4.11	7.02E-06
52	RIMS4	20	43438421, 43438428, 43438430, 43438433	24.21±1.10	41.31±4.04	1.05E-05

53	RIMS4	20	43438452	15.54±1.11	30.29±3.61	6.01E-05
54	RIMS4	20	43438513	9.22±0.60	21.83±3.27	6.05E-06
55	RIMS4	20	43438548, 43438550	3.73±0.43	20.11±4.71	7.83E-05
56	RIMS4	20	43438576	8.44±0.71	27.00±4.47	9.52E-07
57	RIMS4	20	43438596	13.58±0.96	28.97±4.06	8.19E-06
58	RIMS4	20	43438621	10.00±0.48	18.75±1.97	7.83E-07
59	RIMS4	20	43438653	16.26±1.49	42.40±8.63	8.35E-03
60	PCDHAC1	5	140305713	18.47±0.44	24.28±1.64	1.21E-04
61	PCDHAC1	5	140305728, 140305730	14.77±0.83	25.74±3.99	9.14E-03
62	PCDHAC1	5	140305755, 140305757	14.77±0.83	25.74±3.99	9.14E-03
63	PCDHAC1	5	140305769	12.14±0.55	26.74±2.98	1.78E-06
64	PCDHAC1	5	140305821	9.72±0.48	18.09±2.14	1.45E-05
65	PCDHAC1	5	140305827	18.47±0.44	24.28±1.64	1.21E-04
66	PCDHAC1	5	140305870	7.93±0.64	16.64±2.44	1.22E-04
67	PCDHAC1	5	140305880, 140305889, 140305892	20.94±0.95	35.03±3.84	1.37E-04
68	PCDHAC1	5	140305902, 140305905	12.66±0.62	24.97±3.94	2.39E-04
69	PCDHAC1	5	140305916, 140305919	8.50±0.73	22.29±4.38	7.44E-04
70	PCDHAC1	5	140305926	11.81±0.90	22.05±3.02	3.71E-04
71	PCDHAC1	5	140305929, 140305931, 140305933	11.90±0.70	24.86±3.85	1.19E-04
72	PCDHAC1	5	140305947	13.60±1.14	27.74±4.73	1.82E-03
73	PCDHAC1	5	140305994	15.50±0.96	26.30±2.52	3.48E-05
74	PRAC	17	46799645, 46799648	26.96±1.00	51.10±3.56	1.10E-07
75	PRAC	17	46799654	22.88±0.95	47.38±4.37	6.53E-07
76	PRAC	17	46799722	16.39±0.93	38.45±4.47	3.25E-06
77	PRAC	17	46799745	21.84±1.27	49.24±3.90	1.01E-07
78	PRAC	17	46799755	25.52±1.03	47.68±2.26	4.36E-08
79	TRH8	3	129693350, 129693352, 129693355, 129693358	12.02±0.53	22.85±1.89	8.78E-07
80	TRH8	3	129693370	19.46±1.04	33.65±2.25	1.10E-05
81	TRH8	3	129693406, 129693412	21.79±0.78	40.64±1.61	1.27E-08
82	TRH8	3	129693425	12.20±0.60	24.44±1.88	4.01E-07
83	TRH8	3	129693500	13.98±0.58	23.03±0.87	9.44E-07
84	TRH8	3	129693513	5.93±0.65	14.53±2.48	1.14E-04
85	TRH8	3	129693518, 129693521, 129693528	14.06±0.69	29.51±1.58	5.40E-08
86	TRH8	3	129693540, 129693543	8.48±0.73	26.56±2.36	8.53E-08
87	TRH8	3	129693563	7.94±0.53	21.74±2.52	9.64E-07
88	TRH8	3	129693570, 129693574	11.84±0.58	24.28±1.63	1.34E-07
89	TRH8	3	129693586	5.27±0.45	15.51±1.26	4.59E-08
90	TRH8	3	129693607	12.20±0.60	24.44±1.88	4.01E-07
91	TRH8	3	129693613	4.07±0.51	15.08±2.10	3.06E-07
92	TRH8	3	129693620	26.14±0.60	35.74±1.98	5.05E-06
93	TRH8	3	129693628	8.80±0.58	18.54±1.51	8.98E-08
94	TRH8	3	129693635	4.66±0.39	10.85±1.01	1.86E-08
95	TRH8	3	129693672	11.12±0.63	23.10±1.91	2.52E-07
96	TRH8	3	129693692	23.05±1.32	44.05±4.18	3.35E-05
97	TRH8	3	129693698, 129693705	6.01±0.72	15.33±2.21	6.74E-06
98	SLC13A5	17	6616641, 6616644	7.75±0.62	20.38±3.73	6.20E-06
99	SLC13A5	17	6616653, 6616655, 6616657	20.67±0.55	34.60±2.35	1.31E-07
100	SLC13A5	17	6616672, 6616676	18.24±0.57	29.51±3.83	4.79E-04
101	SLC13A5	17	6616702, 6616705, 6616707	9.02±0.55	25.32±4.17	1.10E-06
102	SLC13A5	17	6616733	4.29±0.21	18.86±4.24	6.88E-09
103	SLC13A5	17	6616751	2.17±0.40	13.26±3.93	2.51E-07
104	SLC13A5	17	6616763, 6616768	14.65±0.47	30.13±3.38	9.22E-08
105	SLC13A5	17	6616812	12.85±0.45	27.89±1.67	7.27E-09
106	SLC13A5	17	6616826, 6616828	16.27±0.64	31.43±2.72	6.53E-08
107	SLC13A5	17	6616851, 6616854, 6616857	10.67±0.48	25.61±3.24	5.19E-08
108	SLC13A5	17	6616867	6.86±0.72	20.70±3.76	2.10E-06
109	SLC13A5	17	6616883, 6616888	11.75±0.65	25.86±2.83	1.66E-06

110	SLC13A5	17	6616927, 6616929	16.27±0.64	31.43±2.72	6.53E-08
111	SLC13A5	17	6616949, 6616955	25.24±0.97	38.06±2.81	6.41E-05
112	SLC13A5	17	6616968, 6616973	10.77±0.53	23.20±2.81	3.10E-07
113	SLC13A5	17	6616986, 6616991	23.36±0.58	33.88±2.86	4.46E-05
114	SLC13A5	17	6617030, 6617038, 6617040, 6617044	20.53±0.72	37.68±2.32	1.15E-07
115	SLC13A5	17	6617077	7.74±0.61	25.17±4.97	1.03E-07
116	SLC13A5	17	6617124	23.26±1.18	47.00±4.00	6.53E-07
117	SLC13A5	17	6617192, 6617197, 6617199, 6617206	26.14±1.13	43.03±3.79	2.28E-04
118	SLC13A5	17	6617251, 6617255	17.04±0.90	35.65±3.99	9.49E-07
119	SLC13A5	17	6617287, 6617291	20.64±0.89	36.85±2.77	1.72E-07
120	SLC13A5	17	6617300, 6617305	22.68±1.01	38.87±2.03	4.70E-07
121	SLC13A5	17	6617318	19.86±1.17	37.90±3.70	5.08E-06
122	SLC13A5	17	6617322	14.47±1.02	23.43±2.38	6.20E-03
123	SLC13A5	17	6617368, 6617370	22.35±1.28	39.23±3.11	3.28E-05
124	SLC13A5	17	6617382	15.04±0.94	32.82±3.39	1.24E-06
125	SLC13A5	17	6617392	17.75±0.99	31.33±2.79	1.15E-05
126	SLC13A5	17	6617398, 6617402, 6617405	12.05±0.64	36.96±6.54	7.33E-07
127	SLC13A5	17	6617415	19.66±1.66	48.94±6.41	1.06E-05
128	SLC13A5	17	6617421, 6617423	23.96±1.28	44.10±3.65	1.63E-07
129	SLC13A5	17	6617456	21.09±1.61	42.63±4.30	2.72E-06
130	SLC13A5	17	6617466, 6617470	17.14±1.06	37.53±3.13	2.11E-07
131	SLC13A5	17	6617523	62.77±2.55	75.39±5.03	1.49E-02
132	SLC13A5	17	6617538	45.02±1.26	50.99±2.70	2.09E-02
133	SLC13A5	17	6617559	45.02±1.26	50.99±2.70	2.09E-02
134	SLC13A5	17	6617580	49.89±2.25	66.70±3.08	7.66E-04
135	SLC13A5	17	6617595, 6617597	56.81±1.25	72.50±1.47	2.35E-07
136	ZNF671	19	58238711	7.69±0.34	27.48±4.78	2.46E-06
137	ZNF671	19	58238717	11.68±0.41	25.71±4.28	7.93E-06
138	ZNF671	19	58238740	4.30±0.34	22.57±5.55	1.09E-06
139	ZNF671	19	58238760, 58238762, 58238764	5.02±0.36	26.55±5.77	2.28E-06
140	ZNF671	19	58238771	4.90±0.38	26.26±6.22	2.97E-06
141	ZNF671	19	58238780	10.89±0.40	25.45±4.26	5.17E-08
142	ZNF671	19	58238791, 58238797, 58238799	5.13±0.36	24.56±6.47	5.82E-05
143	ZNF671	19	58238810	5.12±0.30	23.08±5.70	3.09E-07
144	ZNF671	19	58238816	11.68±0.41	25.71±4.28	7.93E-06
145	ZNF671	19	58238823, 58238828	3.59±0.22	14.71±3.40	2.18E-06
146	ZNF671	19	58238832	6.47±0.44	27.74±5.78	1.97E-06
147	ZNF671	19	58238850	8.33±0.47	29.83±5.93	3.40E-07
148	ZNF671	19	58238870, 58238872, 58238877	13.78±0.30	24.95±4.04	2.08E-04
149	ZNF671	19	58238903, 58238905	12.29±0.94	35.04±5.67	1.85E-06
150	ZNF671	19	58238928	6.55±0.36	27.52±5.07	2.38E-08
151	ZNF671	19	58238941	7.69±0.34	27.48±4.78	2.46E-06
152	ZNF671	19	58238954	10.89±0.40	25.45±4.26	5.17E-08
153	ZNF671	19	58238987	10.89±0.40	25.45±4.26	5.17E-08
154	ZNF671	19	58239003	11.68±0.41	25.71±4.28	7.93E-06
155	ZNF671	19	58239012	6.97±0.28	19.12±5.30	6.25E-08
156	ZNF671	19	58239027	7.85±0.26	20.50±5.15	9.26E-07
157	WNT3A	1	228195591	1.78±0.24	5.00±1.20	1.16E-03
158	WNT3A	1	228195628, 228195638	23.54±1.00	37.86±3.41	4.99E-05
159	WNT3A	1	228195659, 228195661, 228195666	17.60±1.11	27.25±3.69	4.64E-03
160	WNT3A	1	228195688	16.72±0.46	31.26±3.04	1.08E-07
161	WNT3A	1	228195722	16.72±0.46	31.26±3.04	1.08E-07
162	WNT3A	1	228195779	16.72±0.46	31.26±3.04	1.08E-07
163	WNT3A	1	228195794	13.88±0.67	26.19±4.15	9.78E-06
164	WNT3A	1	228195848	11.30±0.98	25.45±3.85	7.34E-05
165	WNT3A	1	228195859	9.04±0.86	25.67±4.62	2.80E-05
166	KHDRBS2	6	62996200	7.18±0.63	16.86±2.71	9.29E-05

167	KHDRBS2	6	62996242	15.90±1.08	24.64±2.83	1.02E-03
168	KHDRBS2	6	62996255, 62996265, 62996267	22.04±0.91	32.71±3.21	6.87E-04
169	KHDRBS2	6	62996290	15.86±0.80	24.71±2.74	6.04E-04
170	KHDRBS2	6	62996304, 62996306	24.88±1.26	33.79±3.17	8.40E-03
171	KHDRBS2	6	62996321	20.62±0.92	29.14±2.40	2.42E-03
172	KHDRBS2	6	62996324, 62996327	16.21±0.82	27.07±2.74	1.56E-04
173	KHDRBS2	6	62996334	20.62±0.92	29.14±2.40	2.42E-03
174	KHDRBS2	6	62996380, 62996383, 62996385, 62996387	24.30±1.13	29.79±3.18	1.05E-01
175	KHDRBS2	6	62996401, 62996403	16.21±0.82	27.07±2.74	1.56E-04
176	KHDRBS2	6	62996414	16.42±0.94	28.43±3.06	9.86E-05
177	KHDRBS2	6	62996436, 62996440	22.24±0.95	30.43±2.67	3.02E-03
178	KHDRBS2	6	62996456, 62996458, 62996463, 62996467	20.69±1.11	27.86±3.12	1.33E-02
179	KHDRBS2	6	62996478	16.37±0.93	21.71±2.18	5.03E-02
180	KHDRBS2	6	62996485, 62996487	14.76±0.93	24.93±3.43	8.65E-04
181	KHDRBS2	6	62996561, 62996564, 62996566	21.58±1.14	28.64±3.58	3.66E-02
182	ASCL2	11	2292525	10.10±1.31	26.29±5.81	7.04E-03
183	ASCL2	11	2292538	16.46±0.82	35.93±3.50	3.18E-06
184	ASCL2	11	2292542, 2292544	25.37±0.71	40.00±2.11	8.35E-07
185	ASCL2	11	2292558	16.83±0.69	27.64±2.17	2.36E-05
186	ASCL2	11	2292588	24.73±1.18	40.36±3.32	2.56E-04
187	ASCL2	11	2292602	19.17±0.88	32.43±2.33	1.54E-05
188	ASCL2	11	2292625	2.39±0.18	7.21±1.69	1.66E-03
189	ASCL2	11	2292636	6.33±0.61	16.93±2.69	1.19E-04
190	ASCL2	11	2292658, 2292663	19.77±0.54	29.86±2.03	1.48E-05
191	ASCL2	11	2292701, 2292703	35.23±0.89	49.43±3.24	3.52E-06
192	ASCL2	11	2292751	43.63±1.70	43.79±4.25	9.26E-01
193	ASCL2	11	2292764	24.61±0.78	30.79±2.72	2.10E-02

[a]National Center for Biotechnology Information (NCBI) Database (Genome Build 37). [b]Mann-Whitney *U* test. *P* values of <0.05 are underlined.
