# Supplementary material for: Prognostication of patients with clear cell renal cell carcinomas based on quantification of DNA methylation levels of CpG island methylator phenotype marker genes
Source: BMC Cancer. 2014 Oct 20;14:772. doi: 10.1186/1471-2407-14-772 (PMC4216836; doi:10.1186/1471-2407-14-772)
Supplement: Supplementary file 8 — Additional file 8: Table S6: Eighty-six CpG units showing area under the curve (AUC) values larger than 0.9 in receiver operating characteristic curve analysis for discrimination of CpG island methylator phenotype (CIMP)-positive clear cell renal cell carcinomas (ccRCCs) from CIMP-negative ccRCCs in the learning cohort. (PDF 40 KB) [file 12885_2014_4959_MOESM8_ESM.pdf]

Table S6. Eighty-six CpG units showing area under the curve (AUC) values larger than 0.9 in receiver operating characteristic curve analysis for discrimination of CpG island methylator phenotype (CIMP)-positive clear cell renal cell carcinomas (ccRCCs) from CIMP-negative ccRCCs in the learning cohort.

ID of CpG unit	Gene symbol	Chromo-some	Position of CpG site[a]	AUC value
1	FAM150A	8	58478309	0.936
2	FAM150A	8	58478316, 58478323	0.947
3	FAM150A	8	58478361, 58478363, 58478366	0.912
5	FAM150A	8	58478396, 58478403	0.945
7	FAM150A	8	58478426, 58478428	0.934
8	FAM150A	8	58478477	0.968
9	FAM150A	8	58478496, 58478499	0.939
10	FAM150A	8	58478504	0.911
11	FAM150A	8	58478511	0.968
12	FAM150A	8	58478536	0.925
14	FAM150A	8	58478585, 58478588, 58478592	0.912
16	FAM150A	8	58478624, 58478626	0.939
18	GRM6	5	178422320, 178422324	0.903
19	GRM6	5	178422375, 178422380	0.931
21	ZFP42	4	188916875	0.917
22	ZFP42	4	188916899	0.933
23	ZFP42	4	188916913	0.928
25	ZFP42	4	188916982, 188916984	0.932
28	ZNF540	19	38042472, 38042474	0.928
30	ZNF540	19	38042496	0.983
32	ZNF540	19	38042518	0.960
33	ZNF540	19	38042530, 38042532	0.991
34	ZNF540	19	38042544, 38042552	0.927
36	ZNF540	19	38042576	0.920
41	ZNF540	19	38042800, 38042802	0.941
42	ZNF540	19	38042816	0.928
43	ZNF154	19	58220567	0.956
44	ZNF154	19	58220627	0.966
45	ZNF154	19	58220657, 58220662	0.959
46	ZNF154	19	58220706	0.912
48	ZNF154	19	58220766, 58220773	0.917
56	RIMS4	20	43438576	0.913
58	RIMS4	20	43438621	0.914
74	PRAC	17	46799645, 46799648	0.943
75	PRAC	17	46799654	0.915
77	PRAC	17	46799745	0.944
78	PRAC	17	46799755	0.957
79	TRH	3	129693350, 129693352, 129693355, 129693358	0.903
81	TRH	3	129693406, 129693412	0.973
82	TRH	3	129693425	0.917
83	TRH	3	129693500	0.902
85	TRH	3	129693518, 129693521, 129693528	0.950
86	TRH	3	129693540, 129693543	0.943
87	TRH	3	129693563	0.902
88	TRH	3	129693570, 129693574	0.935

89	TRH	3	129693586	0.952
90	TRH	3	129693607	0.917
91	TRH	3	129693613	0.921
93	TRH	3	129693628	0.943
94	TRH	3	129693635	0.967
95	TRH	3	129693672	0.925
99	SLC13A5	17	6616653, 6616655, 6616657	0.940
101	SLC13A5	17	6616702, 6616705, 6616707	0.906
102	SLC13A5	17	6616733	0.983
103	SLC13A5	17	6616751	0.928
104	SLC13A5	17	6616763, 6616768	0.946
105	SLC13A5	17	6616812	0.983
106	SLC13A5	17	6616826, 6616828	0.951
107	SLC13A5	17	6616851, 6616854, 6616857	0.954
110	SLC13A5	17	6616927, 6616929	0.951
112	SLC13A5	17	6616968, 6616973	0.927
114	SLC13A5	17	6617030, 6617038, 6617040, 6617044	0.942
115	SLC13A5	17	6617077	0.927
116	SLC13A5	17	6617124	0.930
118	SLC13A5	17	6617251, 6617255	0.916
119	SLC13A5	17	6617287, 6617291	0.931
120	SLC13A5	17	6617300, 6617305	0.930
124	SLC13A5	17	6617382	0.942
126	SLC13A5	17	6617398, 6617402, 6617405	0.936
127	SLC13A5	17	6617415	0.908
128	SLC13A5	17	6617421, 6617423	0.927
130	SLC13A5	17	6617466, 6617470	0.935
135	SLC13A5	17	6617595, 6617597	0.942
138	ZNF671	19	58238740	0.906
141	ZNF671	19	58238780	0.954
143	ZNF671	19	58238810	0.926
147	ZNF671	19	58238850	0.927
150	ZNF671	19	58238928	0.965
152	ZNF671	19	58238954	0.954
153	ZNF671	19	58238987	0.954
155	ZNF671	19	58239012	0.951
156	ZNF671	19	58239027	0.910
160	WNT3A	1	228195688	0.943
161	WNT3A	1	228195722	0.943
162	WNT3A	1	228195779	0.943
184	ASCL2	11	2292542, 2292544	0.907

[a]National Center for Biotechnology Information (NCBI) Database (Genome Build 37).
