# Supplementary material for: Prognostication of patients with clear cell renal cell carcinomas based on quantification of DNA methylation levels of CpG island methylator phenotype marker genes
Source: BMC Cancer. 2014 Oct 20;14:772. doi: 10.1186/1471-2407-14-772 (PMC4216836; doi:10.1186/1471-2407-14-772)
Supplement: Supplementary file 9 — Additional file 9: Figure S3: Kaplan–Meier survival curves of patients with CIMP-positive and -negative high-grade (grades 3 and 4) and high-stage (stages III and IV) clear cell renal cell carcinomas (ccRCCs) in the validation cohort. The cancer-free (Panel A, P = 1.01 × 10^−3^) and overall (Panel B, P = 7.04 × 10^−4^) survival rates of patients with CIMP-positive grade 3/4 ccRCCs were significantly lower than those of patients with CIMP-negative grade 3/4 ccRCCs (log-rank test). The cancer-free (Panel C, P = 7.76 × 10^−4^) and overall (Panel D, P = 5.48 × 10^−5^) survival rates of patients with CIMP-positive stage III/IV ccRCCs were significantly lower than those of patients with CIMP-negative stage III/V ccRCCs (log-rank test). Patients who underwent curative resection are included in panels A and C. (PDF 94 KB) [file 12885_2014_4959_MOESM9_ESM.pdf]

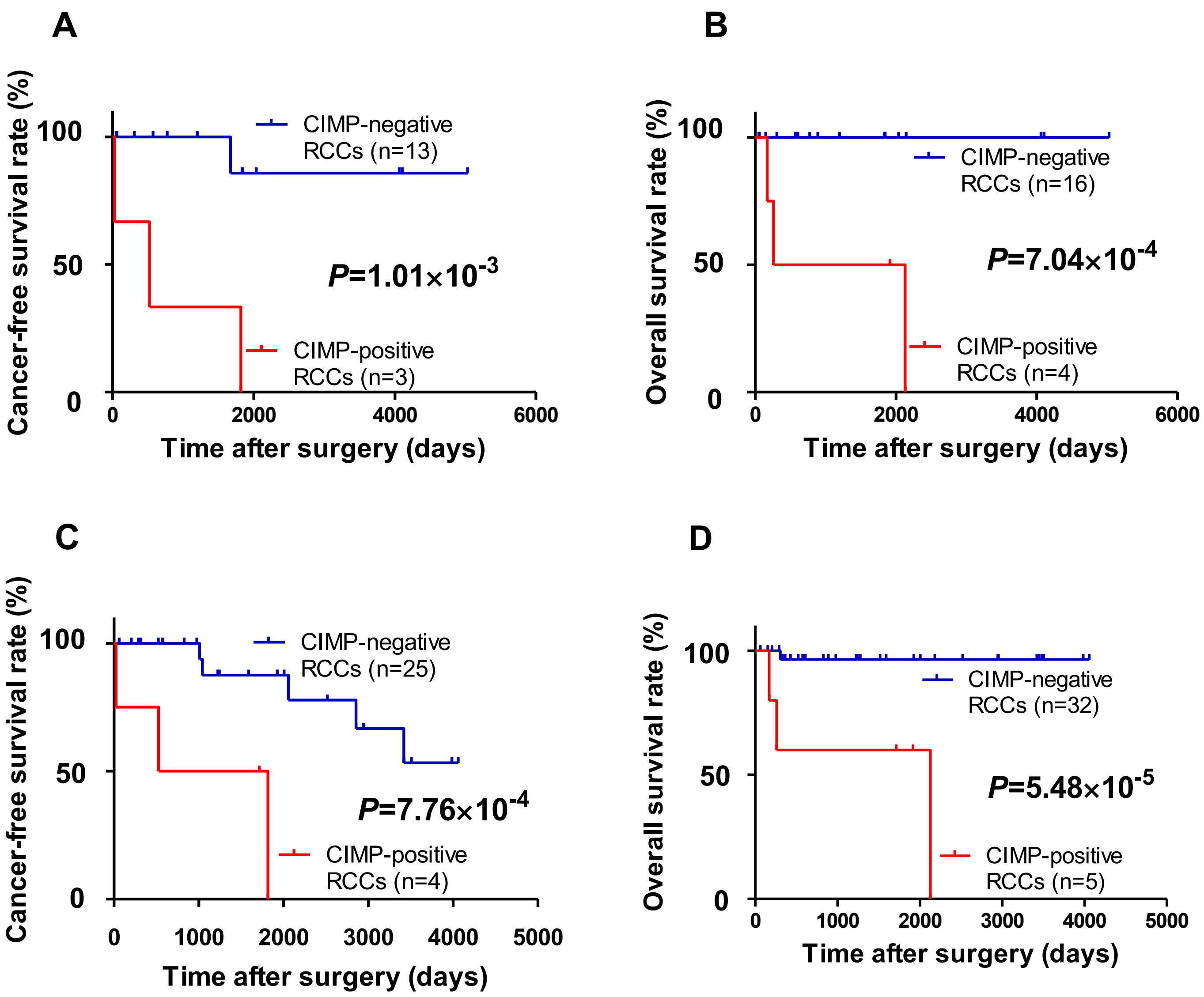

Figure S3. Kaplan–Meier survival curves of patients with CIMP-positive and -negative high-grade (grades 3 and 4) and high-stage (stages III and IV) clear cell renal cell carcinomas (ccRCCs) in the validation cohort. The cancer-free (Panel **A**, P=1.01×10^{-3}) and overall (Panel **B**, P=7.04×10^{-4}) survival rates of patients with CIMP-positive grade 3/4 ccRCCs were significantly lower than those of patients with CIMP-negative grade 3/4 ccRCCs (log-rank test). The cancer-free (Panel **C**, P=7.76×10^{-4}) and overall (Panel **D**, P=5.48×10^{-5}) survival rates of patients with CIMP-positive stage III/IV ccRCCs were significantly lower than those of patients with CIMP-negative stage III/V ccRCCs (log-rank test). Patients who underwent curative resection are included in panels **A** and **C**.
